# Comparative efficacy of digital health intervention delivery modalities on quality of life, anxiety, and depression in coronary heart disease: a systematic review and network meta-analysis

**DOI:** 10.3389/fpsyt.2026.1912762

**Published:** 2026-09-28

**Authors:** Yiran Zhang, Tianyou Yu, Wenrui Huang, Lin Tian, Mutong Li, Jiangbo Wang, Zhaofeng Li

**Affiliations:** 1 College of Acupuncture and Tuina, Shandong University of Traditional Chinese Medicine, Jinan, Shandong, China; 2 The Fourth Clinical Medical College, Guangzhou University of Chinese Medicine, Shenzhen Traditional Chinese Medicine (TCM) Hospital, Shenzhen, Guangdong, China; 3 Shandong University of Traditional Chinese Medicine, Jinan, Shandong, China

**Keywords:** anxiety, cardiac rehabilitation, coronary heart disease, depression, digital health, eHealth, mHealth, quality of life

## Abstract

**Background:**

Nonadherence to traditional cardiac rehabilitation and persistent psychological distress among patients with coronary heart disease (CHD) severely undermine desired therapeutic outcomes. Digital health interventions (DHIs) emerge as promising strategies to effectively tackle this adherence and mental health issue remotely.

**Objective:**

The aim of this study was to conduct a network meta-analysis (NMA) to compare and rank the comparative efficacy of various DHI delivery modalities in improving quality of life (QoL), anxiety, and depression among patients with CHD.

**Methods:**

A systematic search strategy was conducted in PubMed, Embase, Web of Science, Cochrane, CNKI, VIP, WanFang, and SinoMed databases to search for randomized controlled trials (RCTs)published from their inception to February 1,2026.We carried out a frequentist NMA using Stata 18.0to compare the efficacy of various DHIs against standard care. The quality of literature was assessed using the Cochrane Risk of Bias tool (version2.0).The certainty of evidence was evaluated using the Confidence in Network Meta-Analysis (CINeMA) framework, and interventions were ranked utilizing the surface under the cumulative ranking (SUCRA)curve.

**Results:**

A total of 44RCTs involving 8,495 patients were enrolled. The NMA revealed that for improving QoL, telephone follow-up (standardized mean difference 2.98, 95%CI 1.75-4.20) and social media (SMD 1.13, 95%CI 0.42-1.84) were statistically superior to usual care. Notably, telephone follow-up, with a SUCRA of 99.5%, appeared to be the most effective option. Regarding psychological outcomes, web-based platforms demonstrated the highest probability of being the best intervention for reducing both anxiety (SMD -1.56,95%CI-2.34 to -0.79;SUCRA 98.6%) and depression (SMD-1.07,95%CI-1.94to-0.20;SUCRA 91.4%).Safety analyses showed interactive modalities significantly reduced adverse events, whereas one-way SMS carried a significantly higher risk than interactive telephone support(OR 3.38,95%CI 1.13-10.13).

**Conclusions:**

The research suggests that telephone follow-up ranked highest among the included studies for improving short-term QoL, while web-based platforms showed a high probability of being effective for alleviating long-term anxiety and depression. However, these rankings should be interpreted cautiously, as intervention content, duration, and patient populations varied considerably across trials, and the small number of studies for certain modalities limits the robustness of direct comparisons. Social media interventions also emerged as a versatile option with benefits across all three outcomes, though their modest effect sizes warrant further optimization of engagement strategies. Passive SMS interventions present potential safety concerns, as their unidirectional nature may attenuate timely symptom recognition and delay appropriate care-seeking—a hypothesis supported by their significantly higher adverse event rates compared with interactive modalities. These results provide crucial evidence-based support for healthcare providers to tailor specific DHIs to individual patient needs.

**Systematic review registration:**

https://www.crd.york.ac.uk/PROSPERO/, identifier CRD420261380157.

## Introduction

Heart disease, specifically coronary heart disease (CHD), remains a leading cause of death worldwide. In 2021, cardiovascular diseases (CVD) caused approximately 19.8 million deaths—representing approximately 32% of all global mortality and 38% of premature deaths from chronic conditions (1). Within this broader health crisis, CHD alone is responsible for roughly 8.99 million deaths and currently affects an estimated 254 million people globally (2).The impact of CHD extends far beyond physical health, significantly diminishing patients’ overall QoL and psychological well-being. While managing physical symptoms like chest pain, patients frequently endure a persistent fear of disease recurrence, heart failure, or sudden death. Clinical data shows that 20% to 40% of CHD patients experience anxiety or depression, and this mental and emotional burden often worsens over time. For example, a three-year study found that anxiety rates in these patients grew from 42.6% to 51.1%, while depression rates rose from 33.3% to 43.7% (3).

These high rates of mental health struggles are not a coincidence; they are driven by a biological connection often referred to as the “heart-brain axis.” The chronic stress of living with a severe illness disrupts the body’s nervous and hormonal systems. This disruption can cause an overactive stress response and chronically high cortisol levels. Over time, these imbalances trigger widespread inflammation, blood vessel damage, and an increased risk of dangerous irregular heartbeats or blocked arteries. Ultimately, worsening physical health deepens psychological distress, creating a vicious cycle where mental and physical decline constantly reinforce one another (4). Because of this interconnected cycle, traditional cardiac rehabilitation (CR) programs that focus solely on physical metrics and test results are not enough. To truly improve long-term patient outcomes, comprehensive psychological support and mental health management must be fully integrated into standard treatment plans for coronary heart disease.

The treatment of CHD has evolved from relying solely on surgical procedures to embracing comprehensive, long-term strategies that include CR. While CR is proven to reduce the risk of future heart issues, its real-world success is limited by low patient participation. Globally, only 30% to 50% of eligible patients are referred to CR, and very few complete the full program (5). In fact, research shows that over 80% of suitable candidates eventually drop out (6). Beyond practical hurdles like transportation and cost, psychological factors—such as the lack of motivation and social withdrawal linked to anxiety or depression—are major barriers to long-term patient engagement (7).This challenge is compounded by a systemic lack of mental health support in standard cardiac care. Although major guidelines advocate for routine psychological assessments (8), the intense demands of clinical practice often compel physicians to focus primarily on physical metrics, overlooking underlying emotional distress. Furthermore, traditional mental health treatments pose notable drawbacks for cardiovascular patients (9). Pharmacological options often carry arrhythmogenic risks, while in-person psychotherapy is limited by provider shortages, high costs, and pervasive social stigma. Consequently, many CHD patients burdened with anxiety and depression remain underdiagnosed and undertreated, severely eroding their QoL.

Notably, prior systematic reviews and meta-analyses in this field have predominantly centered on clinically-oriented, physical health outcomes—such as major adverse cardiovascular events (MACE), all-cause mortality, blood pressure control, and exercise capacity (10). While these endpoints are undeniably critical, they do not capture the full spectrum of patient wellbeing. The pervasive psychological burden experienced by CHD patients—manifesting as persistent anxiety, depressive symptoms, and severely diminished quality of life (QoL)—has received comparatively less quantitative scrutiny in prior syntheses. Indeed, a recent meta-analysis of internet-based cardiac rehabilitation found no significant reduction in anxiety or depression compared with usual care (11), underscoring the inconclusive and underdeveloped evidence base for psychological outcomes. This disparity is concerning, given that anxiety and depression are not merely secondary comorbidities but independent risk factors for poor cardiovascular prognosis (12), and they fundamentally determine patients’ perceived QoL and long-term adherence to rehabilitation. Therefore, rather than reiterating the well-documented physical benefits of cardiac rehabilitation, the present review explicitly targets these often-overlooked patient-important outcomes to address a critical evidence gap left by previous physically-focused syntheses.

Digital Health Interventions (DHIs) offer a highly disruptive solution to bridge these gaps. By utilizing modern communication technologies, DHIs bypass the physical constraints of traditional clinics, allowing structured rehabilitation, remote monitoring, and tailored psychological support to be seamlessly integrated into patients’ daily lives. While recent studies confirm that various digital tools yield significant clinical benefits (3, 13–15), current evidence regarding their comparative effectiveness remains methodologically limited. Previous reviews have frequently aggregated diverse digital modalities into a single, broad category (10), failing to provide the granular insights required to formulate customized clinical guidelines.

To address these ongoing clinical challenges and research gaps, a comprehensive analysis is essential. This study systematically reviews the latest clinical trials from eight major databases to evaluate how digital health tools affect QoL, anxiety, and depression in patients with coronary heart disease. By comparing different digital approaches, we aim to identify the most effective delivery methods, ultimately providing clear, reliable guidance to improve future patient care.

## Methods

The study protocol is registered in the PROSPERO database (CRD420261380157). We conducted and reported this analysis according to the PRISMA-NMA guidelines for network meta-analyses (16).

### Data sources and search strategy

We systematically searched eight databases (PubMed, Web of Science, Embase, Cochrane Library, CNKI, VIP, Wanfang Data, and SinoMed) from their inception to February 1, 2026. Using a mix of standard index terms and keywords, we focused on concepts such as “coronary heart disease,” “digital health,” “quality of life,” “anxiety,” “depression,” and “randomized controlled trials.” We also searched grey literature to ensure comprehensive coverage. **Appendix 1** details the complete search strategy. In addition to database searching, we screened the reference lists of all included studies and relevant systematic reviews to identify any additional eligible trials. Grey literature was searched via OpenGrey and the WHO International Clinical Trials Registry Platform; however, no eligible RCTs were identified from these sources beyond those captured in the primary database search.

### Inclusion and exclusion criteria

We selected studies based on the PICOS framework:

Participants: Clinically stable adults (≥18 years) with a confirmed diagnosis of CHD. This included patients with acute coronary syndrome, acute myocardial infarction, or a history of heart surgeries (PCI or CABG).
Interventions: Digital health interventions delivered via platforms such as websites, mobile apps, telephone, or virtual reality (VR).
Comparison: Standard non-digital care or conventional cardiac rehabilitation.
Outcomes: QoL, anxiety, depression, and adverse event rates. **Appendix 2** defines the specific digital interventions.
Study Design: Randomized controlled trials (RCTs) only. We restricted eligibility to RCTs because they provide the highest level of evidence for comparative effectiveness by minimizing confounding through random allocation and are the gold-standard design for network meta-analysis, which requires assumptions of transitivity and consistency that are best satisfied by RCT-level aggregate data. We acknowledge that the open-label nature inherent to digital health interventions precludes blinding of participants and personnel, which may introduce performance and detection biases. However, we systematically assessed these potential biases using the Cochrane RoB 2.0 tool and incorporated them into the certainty rating via the CINeMA framework, as detailed in the Risk of Bias and Certainty of Evidence section. We included studies in any language, using translation software for non-English and non-Chinese texts.

We excluded studies if they involved patients without CHD, patients with severe co-existing conditions, or those unable to use electronic devices. We also excluded non-RCTs, animal studies, duplicate publications, incomplete datasets, unvalidated questionnaires, and non-peer-reviewed materials like conference abstracts. For the purpose of this review, we classified SMS-based interventions as ‘passive’ when the original trial descriptions indicated that they consisted solely of unidirectional, automated text messages sent to patients without real-time interactive feedback, bidirectional communication, or healthcare provider responsiveness. Conversely, interventions involving scheduled phone calls with active clinician-patient dialogue, or mobile apps/web platforms that incorporated real-time messaging functions and clinician feedback, were categorized as ‘interactive.’ This distinction was predefined in our protocol and applied consistently during data extraction.

### Data selection and extraction

After managing citations and removing duplicates using EndNote 21, two independent reviewers screened the titles and abstracts. They then evaluated the full texts against our inclusion criteria. Data extracted into standard forms included study details, patient demographics, disease subtype, digital intervention format, treatment duration, control group details, and outcome scores. A third reviewer resolved any disagreements. Literature screening and data extraction were facilitated using EndNote 21 for reference management and Excel spreadsheets for standardized data collection. We did not use automated AI screening tools. The inter-rater reliability for modality categorization was assessed using Cohen’s kappa coefficient (κ = 0.87), indicating near-perfect agreement. Disagreements requiring a third reviewer occurred in 6 out of 306 full-text assessments (2.0%). For the few studies that reported combined interventions (e.g., mobile app plus telephone follow-up), we classified them according to their primary delivery modality as explicitly defined by the trial authors, while systematically recording all adjunctive modalities in the intervention description field for transparency. This approach ensured that each study contributed to only one modality node in the network meta-analysis, thereby preserving the statistical independence of comparisons.

### Risk of bias and certainty of evidence assessment

Two independent reviewers evaluated the quality of the included trials using the Cochrane Risk of Bias 2.0 tool (17). They assessed five key areas: the randomization process, deviations from the intended intervention, missing data, outcome measurement, and reporting selection. Studies were categorized overall as having a “low,” “high,” or “some concerns” risk of bias (18). Additionally, we used the Confidence in Network Meta-Analysis (CINeMA) framework to evaluate the overall reliability of the evidence across six domains: within-study bias, reporting bias, indirectness, imprecision, heterogeneity, and incoherence.

### Statistical analysis

We performed the network meta-analysis using Stata 18.0 to compare the effectiveness of different digital interventions against standard care. Following Cochrane guidelines, we used post-intervention data to calculate pooled effect sizes (19).We chose post-intervention data for two primary reasons. First, because random allocation ensures baseline comparability of key prognostic factors across groups, the post-intervention between-group difference provides an unbiased and clinically interpretable estimate of the treatment effect. Second, the majority of included trials did not report the standard deviation of change-from-baseline scores, which is required to compute standardized mean differences for change data; using post-intervention data thus maximized the inclusion of available evidence while minimizing the need for imputation. We confirmed that baseline outcome values were balanced across comparison groups in all included networks, supporting the validity of this approach. Depending on the data type, we calculated Odds Ratios (OR) or Risk Ratios (RR) for categorical outcomes, and Standardized Mean Differences (SMD) or Mean Differences (MD) for continuous outcomes.

We created network graphs to map the comparisons between different interventions and control groups. In these graphs, node size represents the number of participants, and line thickness indicates the volume of direct comparisons. We assessed overall data consistency using the design-by-treatment interaction model (20) and checked local consistency via the node-splitting method (21). To rank the comparative effectiveness of each intervention, we calculated the Surface Under the Cumulative Ranking (SUCRA) curve, where a score closer to 100% indicates a higher probability of success (21). Finally, we used comparison-adjusted funnel plots to check for potential publication bias (22).

## Results

### Literature search and study selection

Our initial database search yielded 7,175 records. After removing 2,466 duplicates, we screened the titles and abstracts of the remaining 4,709 articles, excluding 4,403 irrelevant records. We then conducted a full-text review of the 306 remaining articles. Of these, 262 were excluded due to incompatible interventions (n=63), inappropriate control groups (n=40), mismatched patient populations (n=71), ineligible study designs (n=73), or unavailable full texts (n=15). Ultimately, 44 randomized controlled trials (RCTs) met our criteria and were included in the network meta-analysis (**Figure 1**).

**Figure 1:**
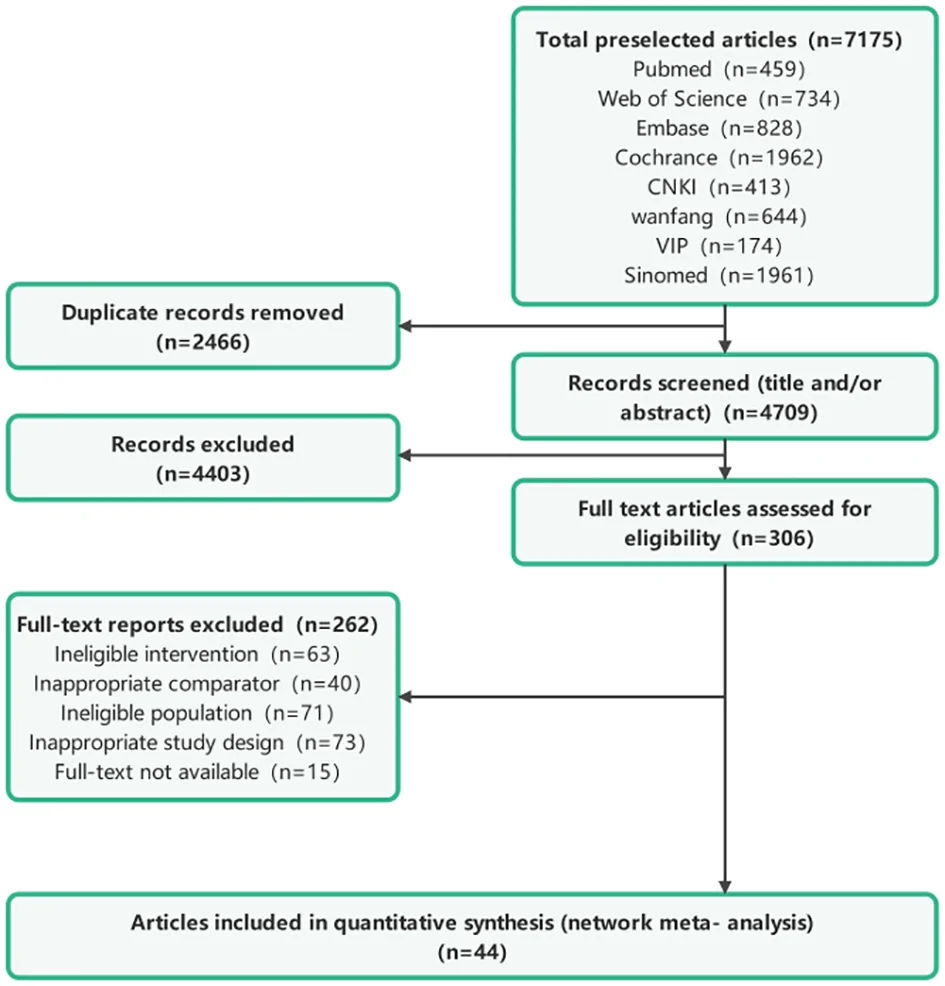
Flowchart of study selection process.

### Characteristics of the included studies

The 44 RCTs included a total of 8,495 participants, with individual study sizes ranging from 26 to 1,424 patients. Participants were primarily male (accounting for 60% to 90% of most cohorts) and generally between 50 and 70 years old. All patients had a confirmed diagnosis of CHD, including acute coronary syndrome, acute myocardial infarction, or a history of surgical interventions like percutaneous coronary intervention or bypass grafting. Treatment durations lasted between three weeks and two years, though most follow-up periods were concentrated between three and six months. We categorized the digital health interventions into seven delivery formats: mobile apps (n=10), social media (n=10), wearables/monitoring (n=10), mobile SMS (n=4), telephone follow-up (n=4), VR (n=4), and web-based platforms (n=2). All control groups received standard care or conventional CR. The detailed characteristics of each included study are presented in **Table 1**.

**Table 1:** Characteristics of included studies.

Study	Country	Duration (months)	Intervention (n)	Control (n)	Outcomes
Reid 2011 (23)	Canada	6	Web-based Platform: Internet-based computer-tailored expert system for physical activity n=223	Usual care n=223	QoL (MacNew)
Dorje 2019 (24)	China	6	Social Media: WeChat-based CR with health education and exercise guidance n=312	Usual care n=312	QoL (SF-12); Anxiety (GAD-7); Depression (PHQ-9)
Snoek 2019 (25)	Netherlands	6	Wearables/Monitoring: Telemonitoring and telecoaching program with activity tracker n=122	Usual care n=122	QoL (MacNew); Anxiety/Depression HADS,PHQ−9
Chow 2022 (26)*	Australia	12	Mobile SMS:4 semi-personalized texts/week on medications and lifestyle;2-way communication with health counselor n=716	Usual care n=708	QoL (SF-12); Anxiety (GAD-7); Depression (PHQ-9)
Vieira 2017 (27)	Portugal	6	VR: Home-based VR exercise program for Phase III CR n=46	Usual care n=46	QoL (MacNew); Anxiety/Depression (DASS-21)
Cheung 2023 (28)	Australia	6	Mobile SMS: Unidirectional texts on self-management support for T2DM/CHD n=506	Usual care n=506	QoL (SF-12); Depression (PHQ-9)
Pitta 2022 (29)	Brazil	3	Telephone Follow-up:Educational program via phone calls n=110	Usual care n=110	Anxiety/Depression (HADS)
Islam 2019 (30)	Australia	6	Mobile SMS: Lifestyle-focused texts (TEXT ME substudy);unidirectional n=710	Usual care n=710	Depression (PHQ-9)
Hisam 2022 (31)	Pakistan	6	Mobile App:mHealth-augmented CR (MCard) n=160	Usual care n=160	QoL (MacNew,SF-12)
Bernal-Jiménez 2024 (32)	Spain	9	Mobile App: EVITE interactive app for lifestyle change post-coronary event n=134	Usual care n=134	QoL (SF-12)
Su 2021 (33)	China	3	Social Media: Nurse-led eHealth CR via WeChat n=146	Usual care n=146	QoL (MacNew); Anxiety/Depression (DASS-21)
Kumar 2024 (34)	India	3	Social Media:eMedia-supported exercise-based Phase II CR n=40	Usual care n=40	QoL (WHOQOL)
Avila 2018 (35)	Belgium	3	Wearables/Monitoring: Home-based telemonitoring-guided rehabilitation n=90	Usual care/Center-based CR n=90	QoL (SF-36)
Fu 2025 (12)	China	6	Web-based Platform: Internet-based continuing nursing with dual heart medical model n=60	Usual care n=60	QoL (SF-36); Anxiety (SAS); Depression (SDS)
Bretschneider 2024 (36)	Germany	1–3	Mobile App: Digital multimodal motivational and educational program n=354	Usual care n=354	QoL (HeartQoL)
Li 2024 (37)	China	12	Social Media: Web-based information-knowledge-attitude-practice intervention via WeChat n=174	Usual care n=174	QoL (SF-12); Anxiety/Depression (HADS)
Hua 2024 (38)	China	1	Telephone Follow-up:Empowerment theory-based phone follow-up post-stenting n=92	Usual care n=92	QoL (SF-36)
Pfaeffli Dale 2015 (39)	New Zealand	6	Mobile SMS: Text4Heart program; daily texts + website; bidirectional for step count feedback n=123	Center-based CR n=123	Anxiety/Depression (HADS)
Gu 2023 (40)	China	3	Social Media: WeChat-based education and rehabilitation post-PCI n=180	Usual care n=180	QoL (EQ-VAS)
Ma 2021 (41)	China	12	Social Media: WeChat-based education and rehab post-CABG for ULMCAD n=140	Usual care n=140	QoL (SF-12); Anxiety/Depression (HADS)
Zou 2024 (42)	China	1.5	Social Media: Mindfulness psycho-behavioral intervention (MCARE) via social media n=178	Usual care n=178	Anxiety (GAD-7); Depression (PHQ-9)
Zhang 2024 (43)	China	24	Social Media: Psycho-cardiological therapy via social media platform n=552	Usual care n=552	QoL EQ−VAS,EQ−5D−5L; Anxiety (GAD-7); Depression (PHQ-9)
Ellis 2025 (44)	Australia	3	Mobile App: Nurse-avatar guided discharge education app n=84	Usual care n=84	QoL EQ−VAS,EQ−5D−5L
Wei 2022 (45)	China	3	Social Media: Structured home-based CR education via WeChat n=80	Usual care n=80	QoL (CQQC)
Hong 2021 (46)	Taiwan, China	3	Wearables/Monitoring: Theory-based health IT intervention with activity tracker n=60	Usual care n=60	QoL (WHOQOL)
Zhang 2020 (47)	China	6	Mobile App: Continuous nursing via mobile medical software n=88	Usual care n=88	Anxiety (SAS); Depression (SDS)
An 2015 (48)	China	3	Telephone Follow-up: Telephone-guided continuous nursing post-PCI n=230	Usual care n=230	QoL (SF-36)
Chen 2017 (49)	China	6	Telephone Follow-up: Telephone follow-up continuous nursing n=150	Usual care n=150	QoL (MLHFQ)
Dalli Peydró 2022 (50)	Spain	10	Wearables/Monitoring: Prolonged mobile care monitoring post-ACS n=67	Center-based CR n=67	QoL EQ−VAS,EQ−5D−5L; Anxiety/Depression (HADS)
Kraal 2017 (51)	Netherlands	3	Wearables/Monitoring: FIT@Home home-based CR with telemonitoring n=90	Center-based CR n=90	QoL (MacNew); Anxiety/Depression (HADS)
Zhang 2025 (52)	China	3	Mobile App: Smartwatch-based home-based CR adherence program n=62	Usual care n=62	QoL (SF-36); Anxiety (GAD-7); Depression (PHQ-9)
Maddison 2019 (53)	New Zealand	3	Wearables/Monitoring: Real-time cardiac telerehabilitation n=162	Center-based CR n=162	QoL (EQ-VAS)
Liu 2026 (54)	China	3	Social Media: eHealth exercise rehab platform based on persuasive systems design n=180	Center-based CR n=180	QoL (SF-36)
Boszko 2023 (55)	Poland	6	Mobile App: After AMI app for QoL and psychological outcomes n=100	Usual care n=100	QoL (MacNew, EQ-VAS)
Bai 2016 (56)	China	12	Mobile App: Android-based cardiac rehabilitation risk assessment software n=150	Usual care n=150	QoL (GQOLI-74)
Batalík 2021 (57)	Czech Republic	3	Wearables/Monitoring: Cardiac telerehabilitation with remote monitoring n=56	Center-based CR n=56	QoL (SF-36)
Liang 2023 (58)	China	3	Mobile App: Exercise program based on mobile medical platform n=95	Usual care n=95	QoL (SF-36); Anxiety (GAD-7); Depression (PHQ-9)
Jóźwik 2021 (59)	Poland	0.75	VR: VR-enhanced cardiac rehabilitation for anxiety/depression n=100	Center-based CR n=100	Anxiety/Depression (HADS)
Li 2025 (60)	China	6	Mobile App: i-CARE smartphone-based CR enhancement n=294	Usual care n=294	QoL (MacNew)
Gong 2025 (61)	China	3	VR: VR technology in early cardiac rehabilitation post-PCI n=92	Usual care n=92	Anxiety (SAS); Depression (SDS)
Zheng 2024 (62)	China	3	Wearables/Monitoring: Home-based cardiac telerehabilitation post-PCI n=110	Usual care n=110	QoL (SF-12)
Fang 2018 (63)	China	1.5	Wearables/Monitoring: Home-based remote sensing CR protocol post-PCI n=80	Usual care n=80	QoL (SF-36); Depression (CDS)
Garcia-Bravo 2020 (64)	Spain	2	VR: VR-based CR for ischemic heart disease n=26	Center-based CR n=26	QoL (SF-36); Depression (Beck-II)
Chong 2025 (65)	Malaysia	3	Wearables/Monitoring: Technology-assisted hybrid CR (TecHCR) n=160	Center-based CR n=160	Anxiety/Depression (HADS)

Full demographic details (age, sex, retention rates, and detailed inclusion/exclusion criteria) are available in **Appendix 2, Supplementary Table 2.1**. QoL, quality of life; CR, cardiac rehabilitation; PCI, percutaneous coronary intervention; CABG, coronary artery bypass grafting; ACS, acute coronary syndrome; ULMCAD, unprotected left main coronary artery disease; T2DM, type 2 diabetes mellitus.

SF-36, Short Form Health Survey-36; SF-12, Short Form Health Survey-12; MacNew, MacNew Heart Disease Health-Related Quality of Life Questionnaire; WHOQOL, World Health Organization Quality of Life; EQ-VAS, EuroQol Visual Analogue Scale; EQ-5D-5L, EuroQol-5 Dimension, 5-Level; HeartQoL, Heart Quality of Life Questionnaire; MLHFQ, Minnesota Living with Heart Failure Questionnaire; CQQC, China Questionnaire of Quality of Life in Patients with Cardiovascular Diseases; GQOLI-74, Generic Quality of Life Inventory-74; GAD-7, Generalized Anxiety Disorder-7; HADS, Hospital Anxiety and Depression Scale; DASS-21, Depression, Anxiety, and Stress Scale-21; SAS, Self-Rating Anxiety Scale; SDS, Self-Rating Depression Scale; PHQ-9, Patient Health Questionnaire-9; Beck-II, Beck Depression Inventory-II; CDS, Cardiac Depression Scale.

†: The TEXTMEDS trial (Chow 2022), while primarily classified as an SMS-based intervention, incorporated a 2-way communication option and a dedicated health counselor who monitored patient replies. This heterogeneity is acknowledged and addressed in the mechanistic interpretation of the safety findings (see Results section ‘Incidence of Adverse Events’), where we note that the inclusion of this partially interactive SMS trial would likely dilute—rather than exaggerate—the observed safety risk estimate for the SMS node.

### Outcome measures

Across the included studies, 20 different measurement tools were used to evaluate participants’ QoL, anxiety, and depression. QoL was measured using 10 different scales, most commonly the SF-36 (n=10), SF-12 (n=8), and the MacNew Questionnaire (n=8). Anxiety was evaluated with standard tools like the HADS-A (n=9) and GAD-7 (n=6), while depression was primarily assessed using the PHQ-9 (n=9) and HADS-D (n=9). A complete breakdown of all clinical assessment tools is available in **Appendix 2; Supplementary Table 2.1**.

### Risk of bias, quality of evidence, and inconsistency

We assessed the methodological quality of the 44 RCTs using the Cochrane Risk of Bias 2.0 tool (**Figure 2**; **Table 2**). Overall, 10 trials (22.7%) were rated as having a low risk of bias, 26 (59.1%) raised some concerns, and 8 (18.2%) were considered high risk. The primary sources of potential bias were related to deviations from intended interventions and how outcomes were measured. Because digital health programs require active participation, blinding patients and researchers is practically impossible. This naturally leads to open-label study designs that inherently raise bias concerns. However, the studies performed well in other areas: the randomization process was generally robust, data was largely complete, and there was little evidence of selective reporting.

**Figure 2:**
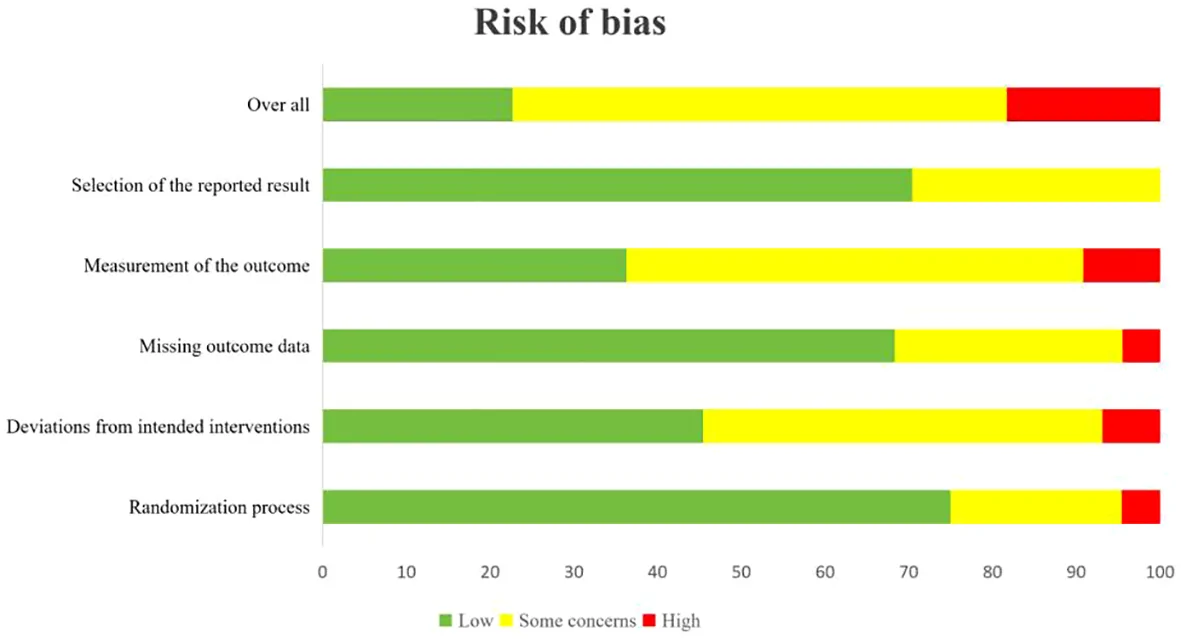
The results of risk of bias assessment.

**Table 2:** Study level risk of bias assessment using Cochrane risk of bias tool 2.0 for assessing risk of bias of randomized clinical trials.

Unique ID	Randomization process	Deviations from intended interventions	Missing outcome data	Measurement of the outcome	Selection of the reported result	Over all
An, 2015 (48)	Low	Some concerns	Low	Some concerns	Some concerns	Some concerns
Avila, 2018 (35)	Low	Low	Low	Some concerns	Low	Some concerns
Bai,2016 (56)	Low	Some concerns	Low	Some concerns	Some concerns	Some concerns
Batalik, 2021 (57)	Low	Some concerns	Some concerns	High	Low	High
Bernal-Jiménez, 2024 (32)	Low	Some concerns	Low	Low	Low	Low
Boszko, 2023 (55)	Low	Some concerns	Some concerns	Low	Low	Some concerns
Bretschneider, 2024 (36)	Low	Some concerns	Some concerns	High	Low	High
Chen,2017 (49)	Some concerns	Some concerns	Low	Some concerns	Some concerns	Some concerns
Cheung, 2023 (28)	Low	Low	Low	Some concerns	Low	Some concerns
Chow 2022 (26)	Low	Low	Low	Some concerns	Low	Some concerns
Dalli Peydró, 2022 (50)	Low	Low	Low	Low	Low	Low
Dorje, 2019 (24)	Low	Low	Low	Low	Low	Low
Ellis, 2025 (44)	Low	Some concerns	Some concerns	Low	Low	Some concerns
Fu, 2025 (12)	Some concerns	Some concerns	Low	Some concerns	Some concerns	Some concerns
Gu, 2023 (40)	Low	Low	Some concerns	Some concerns	Some concerns	Some concerns
Hisam, 2022 (31)	High	Some concerns	High	Low	Low	High
Hong, 2021 (46)	Low	Low	Low	Some concerns	Low	Some concerns
Hua, 2024 (38)	Some concerns	Some concerns	Low	Some concerns	Some concerns	Some concerns
Jóźwik,2021 (59)	Low	High	High	Low	Low	Some concerns
Kraal, 2017 (51)	Low	Low	Low	Some concerns	Low	Some concerns
Kumar, 2024 (34)	Low	Low	Low	Low	Low	Low
Li 2024 (37)	Low	Some concerns	Some concerns	Some concerns	Low	Some concerns
Liang,2023 (58)	Some concerns	Some concerns	Low	Some concerns	Some concerns	Some concerns
Liu, 2026 (54)	Low	Low	Low	Low	Low	Low
Ma, 2021 (41)	Low	Low	Some concerns	High	Some concerns	High
Maddison, 2019 (53)	Low	Low	Low	Low	Low	Low
Pfaeffli Dale 2015 (39)	Low	Low	Low	Some concerns	Low	Some concerns
Pitta, 2022 (29)	Low	Some concerns	Low	Some concerns	Low	Some concerns
Reid, 2011 (23)	Low	Low	Low	Low	Low	Low
Islam, 2019 (30)	Low	Some concerns	Low	Some concerns	Low	Some concerns
Snoek, 2019 (25)	Low	Low	Low	High	Low	High
Su, 2021 (33)	Low	Low	Some concerns	Low	Low	Some concerns
Vieira, 2017 (27)	Low	High	High	Some concerns	Low	High
Wei,2022 (45)	Some concerns	Some concerns	Low	Some concerns	Some concerns	Some concerns
Zhang, 2025 (52)	Low	Some concerns	Low	Some concerns	Low	Some concerns
Zhang,2020 (47)	Some concerns	Some concerns	Low	Some concerns	Some concerns	Some concerns
Zhang,2024 (43)	Some concerns	Some concerns	Low	Some concerns	Low	Some concerns
Zou, 2024 (42)	Low	Low	Low	Low	Low	Low
Li, 2025 (60)	Low	Low	Low	Low	Low	Low
Gong, 2025 (61)	Low	Low	Low	Some concerns	Some concerns	Some concerns
Zheng, 2024 (62)	Some concerns	High	Low	Some concerns	Some concerns	High
Fang, 2018 (63)	High	High	Some concerns	Some concerns	Some concerns	High
García-Bravo, 2020 (64)	Some concerns	Some concerns	Some concerns	Low	Low	Some concerns
Chong, 2025 (65)	Low	Low	Some concerns	Low	Low	Low

Our statistical models showed no significant inconsistency across the network (p≥0.05), successfully validating the transitivity assumption required to compare these interventions. Despite this consistency, the overall certainty of the evidence was rated as “Low” or “Very Low” using the CINeMA framework. This was largely due to the inherent lack of blinding and the small sample sizes in some direct comparisons, which resulted in wide confidence intervals. Finally, funnel plot evaluations showed no significant asymmetry, indicating a low likelihood of publication bias (**Appendices 4, 8, 9**).

### QoL

Our analysis of QoL included 36 trials with 6,841 patients, comparing eight different interventions directly against standard care (**Figure 3**).

**Figure 3:**
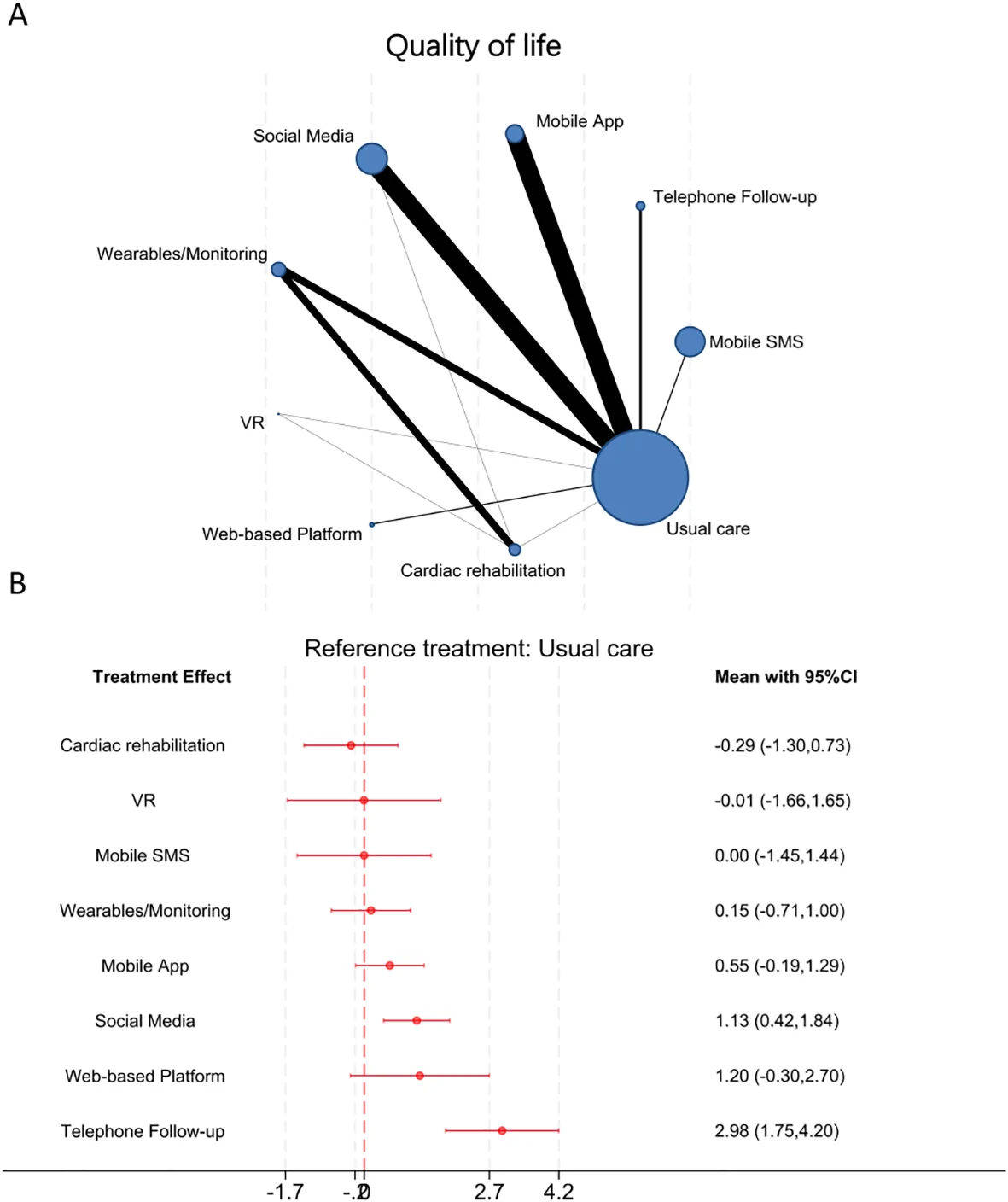
Network map of the effect on QoL. **(A)** Network plot of eligible direct comparisons among intervention modalities for quality of life. Node size is proportional to the total number of participants assigned to each intervention, and line thickness is proportional to the number of studies contributing to each direct comparison. **(B)** Forest plot of standardized mean differences (SMDs) and 95% confidence intervals (CIs) for each intervention versus usual care. After harmonisation of scale direction, positive SMD values indicate better quality of life and favour the intervention.

While most digital interventions showed a general trend toward improving QoL, only two provided statistically significant benefits over standard care. Telephone follow-up demonstrated the greatest overall improvement (SMD 2.98, 95% CI 1.75–4.20, SUCRA 99.5%; Moderate), followed by social media (SMD 1.13, 95% CI 0.42–1.84, SUCRA 76.3%; Moderate). The remaining methods—including web platforms, mobile apps, wearables/monitoring, mobile SMS, VR, and conventional CR—did not show meaningful improvements compared to standard care alone (Appendices 5 and 6).

When comparing the interventions directly against one another, telephone follow-up consistently demonstrated significant advantages over most other modalities. Specifically, it outperformed mobile SMS (SMD 2.98, 95% CI 1.09–4.87), mobile apps (SMD 2.42, 95% CI 0.99–3.85), social media (SMD 1.84, 95% CI 0.43–3.26), wearables/monitoring (SMD 2.83, 95% CI 1.34–4.32), VR (SMD 2.98, 95% CI 0.92–5.04), and conventional CR (SMD 3.26, 95% CI 1.67–4.85) (**Appendix 7; Supplementary Table 7.1**). Additionally, social media provided a significant advantage over conventional rehabilitation (SMD 1.42, 95% CI 0.27–2.56). No other direct comparisons between the remaining interventions showed a significant difference.

### Anxiety

Our analysis of anxiety outcomes included 20 trials involving 4,141 patients, comparing eight interventions directly against standard care (**Figure 4**).

**Figure 4:**
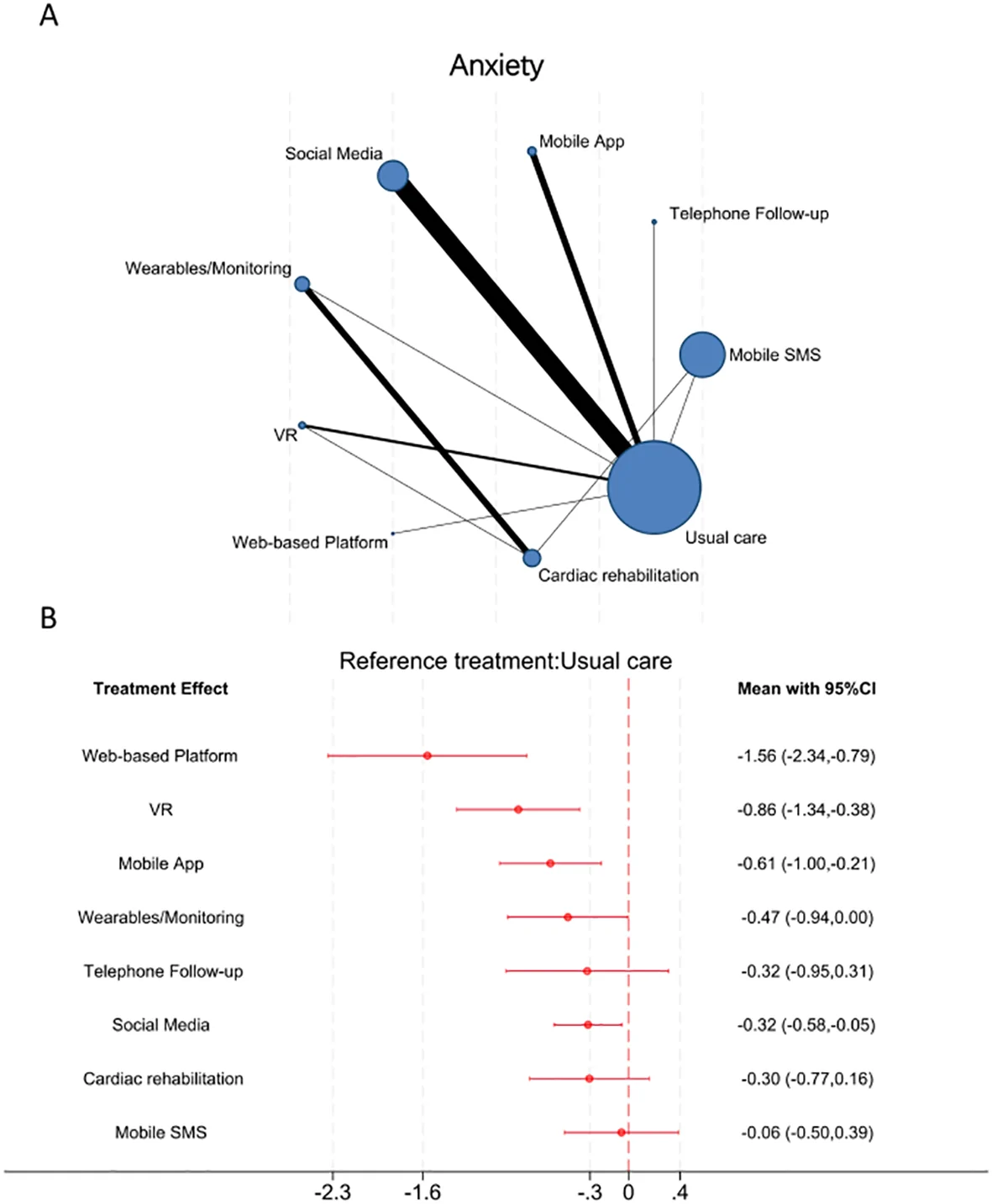
Network map of the effect on Anxiety scores. **(A)** Network plot of eligible direct comparisons among intervention modalities for anxiety. Node size is proportional to the total number of participants assigned to each intervention, and line thickness is proportional to the number of studies contributing to each direct comparison. **(B)** Forest plot of standardized mean differences (SMDs) and 95% confidence intervals (CIs) for each intervention versus usual care. Negative SMD values indicate lower anxiety scores and favour the intervention.

While most digital interventions showed a general trend toward reducing anxiety, only four provided statistically significant improvements over standard care alone. Web-based platforms demonstrated the greatest reduction in anxiety (SMD -1.56, 95% CI -2.34 to -0.79, SUCRA 98.6%;Moderate), followed by VR (SMD -0.86, 95% CI -1.34 to -0.38, SUCRA 82.7%;Moderate), mobile apps (SMD -0.61, 95% CI -1.00 to -0.21, SUCRA 67.1%;Moderate), and social media (SMD -0.32, 95% CI -0.58 to -0.05, SUCRA 40.7%;Low). The other approaches—including wearables/monitoring, telephone follow-up, mobile SMS, and conventional CR—did not significantly reduce anxiety compared to standard care (Appendices 5 and 6).

When comparing the active interventions directly against each other, web-based platforms consistently proved to be the most effective. They significantly outperformed mobile SMS (SMD 1.51, 95% CI 0.62–2.40), telephone follow-up (SMD 1.24, 95% CI 0.24–2.24), mobile apps (SMD 0.96, 95% CI 0.09-1.82), social media (SMD 1.25, 95% CI 0.43–2.07), wearables/monitoring (SMD 1.09, 95% CI 0.19–2.00), and conventional CR (SMD -1.26, 95% CI -2.16–0.36) (**Appendix 7; Supplementary Table 7.2**). Additionally, VR provided a significant advantage over both mobile SMS (SMD 0.80, 95% CI 0.21–1.40) and conventional rehabilitation (SMD -0.55, 95% CI -1.07– 0.03). No other direct comparisons between the remaining interventions showed a significant difference in reducing anxiety.

### Depression

Our analysis of depression outcomes included 24 trials involving 5,463 patients, comparing eight interventions directly against standard care (**Figure 5**).

**Figure 5:**
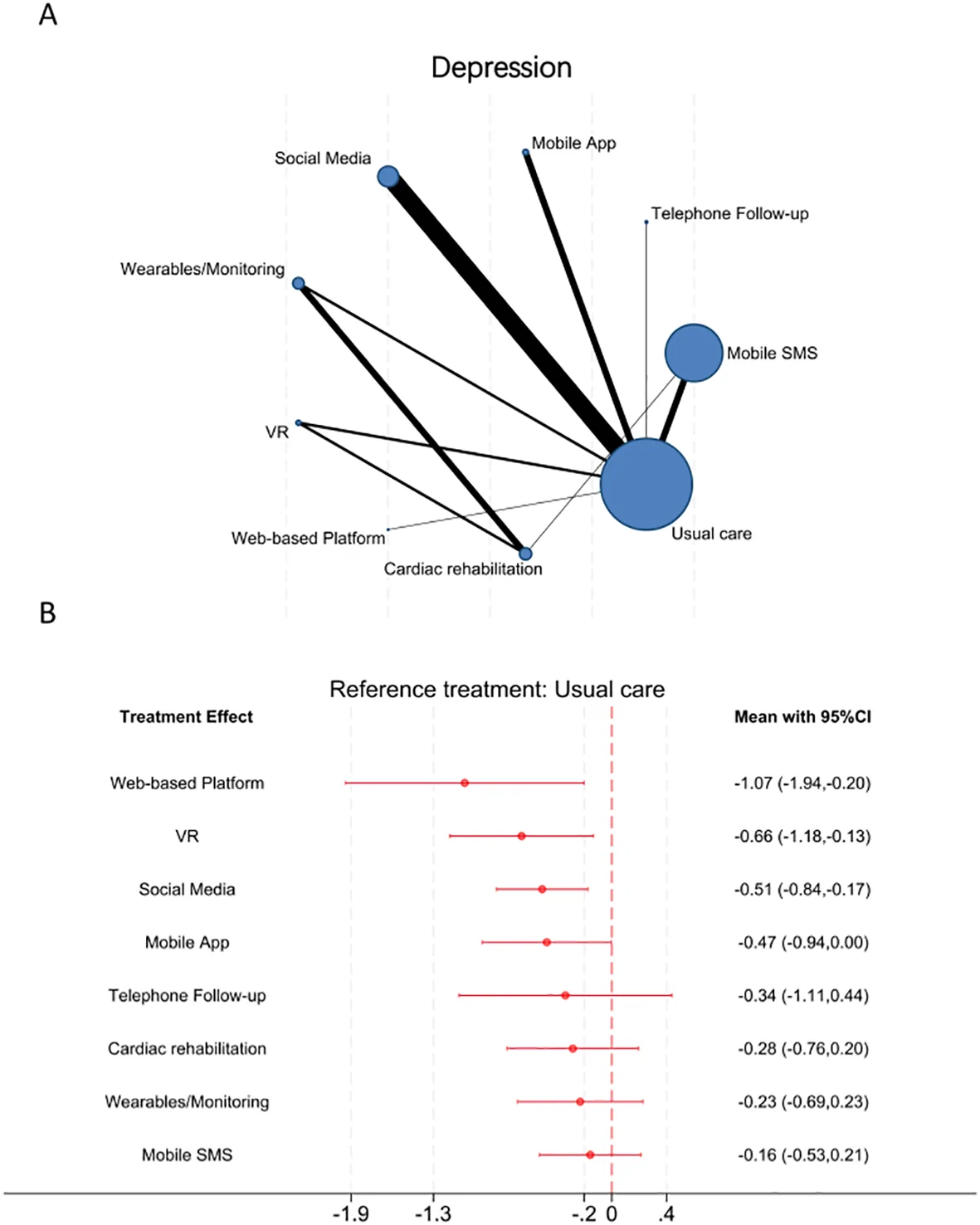
Network map of the effect on depression scores. **(A)** Network plot of eligible direct comparisons among intervention modalities for depressive symptoms. Node size is proportional to the total number of participants assigned to each intervention, and line thickness is proportional to the number of studies contributing to each direct comparison. **(B)** Forest plot of standardized mean differences (SMDs) and 95% confidence intervals (CIs) for each intervention versus usual care. Negative SMD values indicate lower depressive symptom scores and favour the intervention.

Only three digital interventions provided statistically significant improvements in depression compared to standard care alone. Web-based platforms demonstrated the greatest reduction (SMD -1.07, 95% CI -1.94 to -0.20, SUCRA 91.4%; Low), followed by VR (SMD -0.66, 95% CI -1.18 to -0.13, SUCRA 76.5%;Moderate) and social media (SMD -0.51, 95% CI -0.84 to -0.17, SUCRA 64.7%;Low). The other approaches—including mobile apps, telephone follow-up, conventional CR, wearables/monitoring, and mobile SMS—did not significantly improve depression scores (Appendices 5 and 6).

Despite these positive results against standard care, comparing the digital interventions directly to one another revealed no significant differences (**Appendix 7; Supplementary Table 7.3**). While web-based platforms, VR, and social media were superior to standard care, no single digital method proved definitively more effective than the others in alleviating depression.

### Incidence of adverse events

Our analysis of adverse events included 34 trials involving 7,107 patients, comparing eight interventions directly against standard care (**Figure 6**).

**Figure 6:**
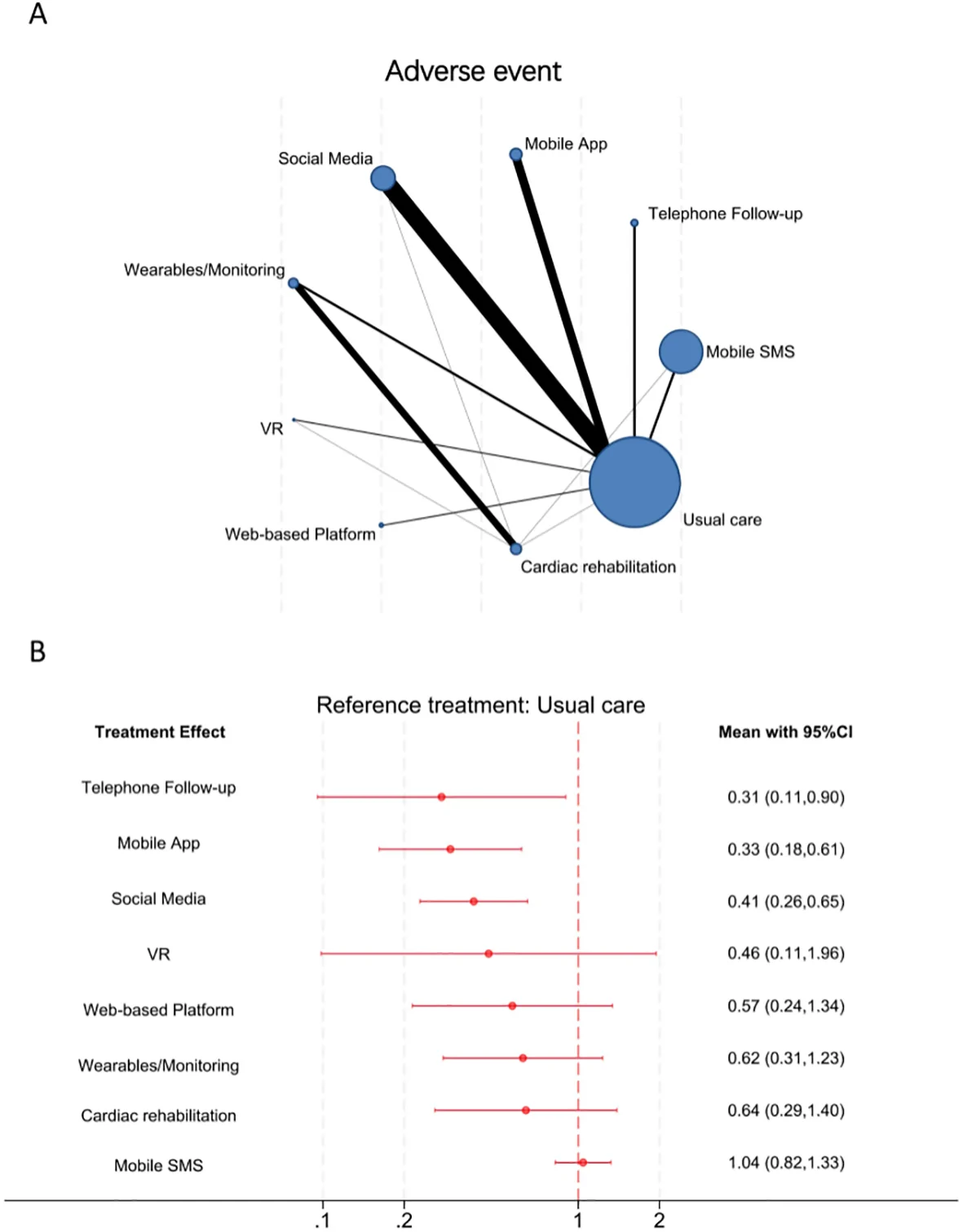
Network map of the effect on adverse events. **(A)** Network plot of eligible direct comparisons among intervention modalities for adverse events. Node size is proportional to the total number of participants assigned to each intervention, and line thickness is proportional to the number of studies contributing to each direct comparison. **(B)** Forest plot of odds ratios (ORs) and 95% confidence intervals (CIs) for each intervention versus usual care. ORs below 1 indicate lower odds of adverse events and favour the intervention.

Compared to standard care alone, three digital interventions significantly reduced the risk of adverse events. Telephone follow-up provided the greatest safety benefit (OR 0.31, 95% CI 0.11–0.90, SUCRA 81.1%; High), closely followed by mobile apps (OR 0.33, 95% CI 0.18–0.61, SUCRA 79.6%;Moderate) and social media (OR 0.41, 95% CI 0.26–0.65, SUCRA 70.7%;High). The other approaches—including VR, web-based platforms, wearables/monitoring, and conventional CR—did not significantly alter the risk of adverse events (Appendices 5 and 6).

When comparing the active interventions directly against each other, mobile SMS carried a significantly higher risk of adverse events than telephone follow-up (OR 3.38, 95% CI 1.13–10.13), mobile apps (OR 3.14, 95% CI 1.62–6.06), and social media (OR 2.56, 95% CI 1.52–4.32) (**Appendix 7; Supplementary Table 7.4**). This indicates that these three more interactive methods offer a much safer alternative to relying solely on mobile SMS.

The adverse events reported across the trials were primarily cardiovascular, including unplanned hospital readmissions, major adverse cardiovascular events (MACE), severe irregular heartbeats, or the need for additional surgeries. A small minority of records noted non-cardiovascular issues, failure to take prescribed medications, or difficulty tolerating specific interventions (such as high dropout rates among older patients using VR). Ultimately, the data confirms that digital health interventions do not increase the risk of severe clinical events, demonstrating a highly robust safety profile for patients with coronary heart disease.

The elevated risk of adverse events associated with SMS-based interventions warrants careful mechanistic interpretation. Importantly, none of the included SMS trials explicitly reported delays in patient-initiated help-seeking or documented cases where delayed SMS replies contributed to missed acute events. However, the significantly higher OR for adverse events in the SMS group (relative to interactive modalities) suggests a clinically meaningful safety deficit. We hypothesize that the unidirectional, non-responsive nature of passive SMS may create a ‘false reassurance’ effect—patients may overestimate their level of active monitoring, potentially attenuating their vigilance for early warning symptoms and delaying emergency help-seeking during acute deterioration. While this mechanistic pathway remains inferential in the absence of direct process data, the observed safety signal from 34 trials underscores that interactive communication channels (telephone, apps, social media) offer superior safety profiles, likely through their capacity for real-time clinical feedback and early detection of red-flag symptoms. Future studies should explicitly capture patient response times and help-seeking behaviors to test this hypothesis.

### Sensitivity analysis

To confirm the reliability of our results, we conducted a sensitivity analysis by excluding studies with a high risk of bias. Removing these studies did not change the primary outcomes for QoL, anxiety, or depression (**Appendix 10; Supplementary Table 10.1**). The interventions that originally showed significant benefits remained effective, proving that our overall findings are highly robust.

### Subgroup analysis

To ascertain the impact of diverse clinical contexts and intervention characteristics on outcomes, we conducted pre-specified subgroup analyses.

#### Intervention content

Programs centered on psychological support provided the greatest improvements in QoL (SMD 1.53, 95% CI 0.52 to 2.54), while exercise-based interventions also yielded substantial benefits for this outcome (SMD 1.14, 95% CI 0.26 to 2.02). For reducing anxiety, exercise-based programs proved to be the most effective strategy (SMD -0.67, 95% CI -1.17 to -0.17), although psychological support (SMD -0.53, 95% CI -0.98 to -0.07) and health tracking (SMD -0.45, 95% CI -0.84 to -0.06) also offered meaningful relief. Conversely, only psychological interventions achieved statistical significance in mitigating depression (SMD -0.59, 95% CI -1.01 to -0.16). These findings highlight that psychological interventions are the premier choice for enhancing QoL and alleviating depression, whereas exercise programs are optimal for managing anxiety (**Appendix 11**).

#### Treatment duration

The effectiveness of these digital tools shifted based on the duration of care. For short-term programs (under six months), telephone follow-up was the most effective method for improving QoL (SMD 2.25, 95% CI 0.45 to 4.06), with social media also demonstrating significant efficacy (SMD 1.70, 95% CI 0.51 to 2.90). Regarding short-term psychological outcomes, VR significantly reduced anxiety (SMD -1.18, 95% CI -2.04 to -0.31), and social media was the sole modality to significantly relieve depression (SMD -0.79, 95% CI -1.52 to -0.06).

For long-term care (six months or more), telephone follow-up continued to yield the strongest improvements in QoL (SMD 4.47, 95% CI 3.14 to 5.81), complemented by significant benefits from web-based platforms (SMD 1.13, 95% CI 0.21 to 2.05) and mobile apps (SMD 0.61, 95% CI 0.06 to 1.17). However, web-based platforms emerged as the strongest long-term strategy for managing both anxiety (SMD -1.56, 95% CI -2.16 to -0.97) and depression (SMD -1.07, 95% CI -1.85 to -0.30). Furthermore, mobile apps were the only intervention that significantly improved long-term patient retention (RR 1.88, 95% CI 1.03 to 3.44). These results suggest that telephone follow-up, VR, and social media are best suited for short-term engagement, whereas web platforms and mobile apps are ideal for long-term management (**Appendix 12**).

#### Economic context

Finally, we evaluated the impact of national income levels on treatment success. In middle-income countries, telephone follow-up significantly boosted QoL (SMD 2.98, 95% CI 1.57 to 4.39), as did social media (SMD 0.90, 95% CI 0.04 to 1.77). In these nations, web platforms were the most effective tools for reducing both anxiety (SMD -1.56, 95% CI -2.29 to -0.84) and depression (SMD -1.07, 95% CI -1.82 to -0.32), followed by sequential benefits from VR, mobile apps, and social media.

Conversely, across high-income countries, none of the digital tools yielded statistically significant improvements in QoL compared to standard care alone in our subgroup analysis (P > 0.05; **Appendix 13**). This pronounced discrepancy may reflect a potential ‘ceiling effect’—because conventional healthcare baselines are already highly advanced in high-income nations, the incremental clinical benefit of single-modality digital interventions may be less apparent than in middle-income regions. However, given the small number of high-income-origin trials and the exclusion of multi-modality interventions from the primary NMA, this observation should be interpreted as hypothesis-generating rather than conclusive.

## Discussion

### Primary outcome

In this network meta-analysis, we systematically evaluated the comparative effectiveness of diverse DHIs in ameliorating QoL, anxiety, and depression among patients with CHD. By synthesizing direct and indirect comparative evidence, we quantitatively ranked multiple digital modalities to identify optimal interventional pathways, thereby establishing a robust foundation for the clinical selection of tailored digital health strategies. Our findings explicitly indicate that while DHIs generally enhance overall patient prognosis, the clinical benefits derived from disparate modalities exhibit profound outcome specificity. For the enhancement of QoL, telephone follow-up and social media interventions demonstrated substantial efficacy, with telephone follow-up ranking highest in the present network, though this finding requires confirmation in larger head-to-head trials. Conversely, in the mitigation of psychological distress—namely anxiety and depression—web-based platforms, VR, and social media exhibited distinct therapeutic advantages. Web-based platforms, in particular, demonstrated exceptional potential for improving comprehensive psychological outcomes.

Furthermore, our results substantiate the robust overall safety profile of DHIs in the longitudinal management of CHD. Notably, interactive modalities—specifically telephone follow-up, mobile applications, and social media—were significantly superior to standalone mobile SMS in mitigating the risk of adverse events. This finding not only dispels lingering concerns that remote digital interventions might inadvertently elevate the risk of cardiovascular events, but also implies that unidirectional information delivery may be fundamentally insufficient to safeguard clinical safety in this vulnerable patient population.

Our findings concurrently underscore the significant moderating influence of intervention module design, treatment duration, and the macroeconomic environment on clinical efficacy. Interventions incorporating dedicated psychological support or exercise guidance modules yielded the most pronounced clinical benefits, affirming the critical importance of multimodal functionality in the architectural development of DHIs. Moreover, the selection of the optimal intervention modality is distinctly time-dependent: securing short-term clinical benefits is best achieved through telephone follow-up and social media, whereas sustained, long-term psychological remodeling necessitates the structural support of web-based platforms.

Finally, current evidence reveals that the significant clinical benefits of DHIs are predominantly concentrated within middle-income countries. In high-income countries, the precise efficacy of single-modality DHIs remains equivocal based on the limited number of available studies originating from these settings. The apparent lack of added benefit in this subgroup may reflect a ceiling effect of already high-standard usual care, rather than the ineffectiveness of the digital interventions per se. Importantly, because our primary analysis focused on modality-level comparisons, and the number of high-income-country trials was small, this finding should be considered exploratory. Future large-scale studies specifically designed for high-income healthcare contexts—and incorporating multi-component or more personalized digital strategies—are needed to determine whether such interventions can overcome this ceiling effect.

Collectively, this heterogeneity in the evidence distribution dictates that the future design and deployment of digital cardiovascular interventions must pivot toward highly individualized, bespoke strategies that meticulously account for patient-specific outcome requirements, intervention trajectories, and the regional allocation of healthcare resources.

### Comparison with previous studies

QoL is a key measure of long-term success in treating coronary heart disease. Our analysis shows that telephone follow-up significantly improves QoL compared to standard care (SMD 2.98, 95% CI 1.75–4.20). This aligns with previous studies highlighting the value of phone-based support (66). Past reviews have evaluated diverse digital interventions collectively as ‘eHealth’ or ‘mHealth’, revealing only modest aggregate benefits (e.g., pooled SMDs ranging from 0.17 to 0.54) (10, 13). In contrast, our specific modality rankings reveal that telephone follow-up achieved the highest SUCRA value (99.5%) for improving QoL. However, this finding relies on just four trials with considerable heterogeneity in intervention protocols, and the confidence intervals around direct comparisons were wide. Therefore, while telephone follow-up appears promising, its apparent superiority over other modalities should be interpreted as hypothesis-generating rather than definitive. Still, direct comparisons showed that telephone support clearly outperformed mobile SMS, mobile apps, VR and conventional rehabilitation. In contrast, automated tools like wearables—which lack human interaction—failed to meaningfully improve QoL (67).

In addition to its effect on QoL, telephone follow-up also contributed to safety outcomes, as discussed below. Beyond physical well-being, digital tools also significantly improved mental health. Web-based platforms, VR, and social media all effectively reduced anxiety and depression compared to standard care, while mobile apps specifically helped alleviate anxiety. Web-based platforms ranked as the most effective tool for managing both anxiety (SUCRA = 98.6%) and depression (SUCRA = 91.4%), closely followed by VR. This supports recent studies on the use of VR in early CR (68). When compared directly against other methods, web platforms were significantly better at reducing anxiety than mobile SMS, phone support, mobile apps, and conventional rehabilitation. This is likely because complex psychological challenges respond better to the deep cognitive restructuring offered by web platforms than to simple physical training programs (69). Social media-based interventions also demonstrated consistent benefits across multiple outcomes, including QoL (SMD 1.13), anxiety (SMD -0.32), and depression (SMD -0.51). This broad-spectrum efficacy likely stems from the peer support and community reinforcement inherent to social media platforms, which may mitigate the social isolation frequently reported by CHD patients. However, the effect sizes for psychological outcomes were modest, and the high variability in platform usage (e.g., WeChat vs. Facebook-based programs) suggests that cultural and technological familiarity may moderate its effectiveness. VR also outperformed mobile SMS and conventional care in reducing anxiety. However, no single digital tool was proven superior for treating depression when compared directly to the others. As with telephone support, the data for web platforms and VR relies on a small number of studies, meaning these strong positive results should be validated further to rule out exaggerated effects.

In terms of patient safety, telephone follow-up (OR 0.31, 95% CI 0.11–0.90), mobile apps (OR 0.33, 95% CI 0.18–0.61), and social media (OR 0.41, 95% CI 0.26–0.65) significantly reduced the risk of adverse clinical events compared to standard care. Conversely, relying solely on mobile SMS carried a much higher risk of adverse events than these more interactive methods. Large trials have shown that one-way mobile SMS can improve basic health metrics like cholesterol and blood pressure (70). However, mobile SMS falls short in preventing severe cardiac events because it is a one-way system without a feedback loop, making it incapable of detecting early warning signs of clinical deterioration (71).

Finally, the success of these digital interventions depends heavily on their specific features, duration, and the broader economic setting. Programs that included dedicated mental health modules provided the greatest improvements in QoL (SMD 1.53) and depression (SMD -0.59). This supports recent meta-analyses demonstrating that psychological eHealth interventions, particularly those incorporating cognitive-behavioral therapy, are vital for alleviating depressive symptoms in cardiovascular patients (72). Timeframe also matters: telephone follow-up (SMD 2.25) and social media (SMD 1.70) delivered the fastest results (under six months). For long-term care (six months or more), web-based platforms were best at maintaining relief from anxiety (SMD -1.56) and depression (SMD -1.07), while mobile apps uniquely improved long-term patient retention (RR 1.88, 95% CI 1.03–3.44). While previous reviews have often reported inconclusive long-term effects of eHealth on QoL and sustained behavioral changes (73), our findings indicate that matching specific digital modalities—such as web platforms for psychological maintenance and apps for adherence—can effectively overcome this temporal decay.

Economically, we observed a distinct “ceiling effect.” Digital tools vastly improved QoL in middle-income countries but showed no significant added benefit over standard care in high-income countries. This aligns precisely with recent evidence showing that mobile health interventions yield significantly greater clinical improvements when implemented in low- or middle-income countries (74). This suggests that the true value of supplemental digital interventions depends heavily on the existing baseline quality of local healthcare resources; in high-income settings, the already optimized standard of care creates a ceiling effect that digital tools struggle to surpass.

An important cross-cutting consideration is the inter-relationship between the psychological outcomes examined in the present review and the physical health outcomes predominantly reported in prior literature. Although our analysis did not directly pool physiological endpoints, the included psychological-focused trials often reported secondary physical outcomes such as blood pressure, lipid profiles, and exercise capacity. Notably, across these studies, improvements in anxiety and depression scores were consistently accompanied by favorable trends in these physical parameters, although formal correlation analyses were rarely performed. This pattern aligns with the broader evidence base: meta-analyses of digital interventions targeting physical outcomes have shown modest but significant reductions in systolic blood pressure and LDL-cholesterol levels (70, 71), while parallel psychological syntheses have independently demonstrated alleviation of depressive symptoms (72). The convergence of these two streams of evidence suggests that DHIs may simultaneously influence both the ‘heart’ and ‘brain’ through interconnected pathways—reducing psychological distress may lower sympathetic nervous system overactivity and systemic inflammation, thereby improving vascular function and reducing cardiac event risk (46).

The clinical implications of this inter-relationship are twofold. First, it reinforces the necessity of integrating psychological assessment into routine cardiac rehabilitation protocols—not as an ancillary add-on, but as a core component that may directly modify physical risk trajectories. Second, it suggests that digital tools should be designed with dual objectives: to deliver structured psychological support while simultaneously tracking behavioral and physiological parameters. Interventions that achieve both—such as web-based platforms combining CBT modules with exercise logging and vital sign monitoring—may offer compounded benefits that exceed those of single-domain approaches. However, caution is warranted: because our NMA focused on modality comparisons rather than direct physiological outcome measurements, the inter-relationship we observe remains inferential. Dedicated head-to-head trials with both psychological and physical endpoints are needed to confirm the extent to which psychological improvements mediate or moderate physical recovery in CHD patients.

### Mechanisms of action

The distinct differences in efficacy and safety among digital health interventions stem from how their underlying technologies interact with patients. For instance, telephone follow-ups are uniquely effective at improving a patient’s QoL because they quickly establish a strong human connection between the patient and the healthcare provider. Older patients with CHD often struggle with low digital literacy, and complex mobile apps can trigger technology-related anxiety. In contrast, telephone calls offer natural verbal communication that provides immediate empathy. This human touch effectively relieves the social isolation and fear of death that patients frequently experience shortly after hospital discharge. Furthermore, phone calls allow clinicians to engage in a continuous, two-way dialogue—asking questions, gathering feedback, explaining instructions, and confirming understanding. This allows providers to immediately adjust rehabilitation guidance based on how well the patient understands it, thereby boosting treatment adherence and patient confidence in ways that automated programs cannot match. Consequently, communication-driven digital strategies that strengthen the clinician-patient relationship are becoming vital tools in personalized cardiovascular care.

While telephone support excels at personal connection, comprehensive web-based platforms offer superior advantages for structured psychological recovery. These platforms can systematically deliver multi-module cognitive behavioral therapy (CBT)—a method shown to significantly reduce cardiovascular mortality in CHD patients when added to standard care. Unlike mobile apps that offer fragmented tips, web platforms allow for self-paced, asynchronous learning. Patients can access mental health education, reframe negative thoughts, and practice coping exercises repeatedly without time constraints, helping them reverse deep-seated harmful mindsets, such as the tendency to catastrophize their illness. Over time, this structured psychological support can physically reduce chronic stress, lower systemic inflammation, and improve blood vessel function, creating a beneficial loop where mental recovery directly aids heart health (75). For faster, short-term anxiety relief, VR offers an alternative approach by deeply engaging the senses. The highly immersive nature of VR occupies the brain’s limited capacity to process sensory information. By blocking out negative bodily sensations, VR temporarily interrupts the cycle of anxiety (76). Its high level of engagement and immersion effectively distracts attention from negative cardiovascular somatic sensations, while progressive exposure in simulated environments facilitates systematic desensitization and cognitive restructuring, specifically targeting kinesiophobia and the fear of recurrent cardiac events (77).

Ultimately, the clinical safety and success of these interventions—whether telephone, apps, or web platforms—depend on whether they allow for two-way communication rather than one-way broadcasting. Interactive, two-way systems equipped with early detection capabilities can immediately alert providers and trigger medical interventions if a patient’s vital signs fluctuate at home, stopping potential cardiovascular crises early. Integrating these responsive systems with artificial intelligence could further strengthen patient safety. On the other hand, simple one-way mobile SMS often cause patients to ignore alerts due to message fatigue. More dangerously, these passive texts can create a false sense of security, leading patients to mistakenly believe they are being constantly monitored 24/7. When early warning signs of an acute cardiac event actually occur, this false confidence can lead to catastrophic delays in seeking emergency medical services (EMS).

### Clinical implications and future directions

This study highlights telephone follow-ups, web platforms, mobile apps, and VR as the most effective digital tools for improving the QoL and mental well-being of patients with CHD. To maximize their impact, clinical practices should integrate these technologies through a phased care plan tailored to a patient’s specific recovery stage. For instance, empathetic telephone calls and immersive VR are highly beneficial in the first six months after hospital discharge to help ease acute anxiety. As patients enter long-term recovery (six months and beyond), transitioning to interactive apps or web platforms can offer the flexibility needed to keep them engaged in ongoing remote care.

However, our findings show that digital tools designed solely for passive health tracking offer limited benefits because they do not actively encourage behavioral changes. Therefore, future digital strategies must move beyond simple monitoring. To achieve meaningful physical and mental recovery, these platforms need to incorporate dedicated, action-oriented features, such as structured exercise programs or targeted psychological support.

Finally, the successful rollout of these interventions depends heavily on broader economic and social factors. In middle-income countries with limited medical resources, digital health tools should be prioritized as a cost-effective way to expand access to care. Conversely, high-income countries must actively address the digital divide to ensure that vulnerable populations—such as those limited by remote locations, financial barriers, or physical disabilities—are not left behind. Ultimately, pairing the right delivery platforms with active, engaging therapies, while respecting regional economic realities, will ensure that future digital healthcare benefits all CHD patients fairly and effectively.

### Strengths and limitations

This study offers the first comprehensive comparison of various digital health interventions for CHD, evaluating both their safety and their impact on QoL and mental health. Unlike previous research that grouped all digital tools together, we directly compared specific technologies to identify the most effective options for different patient outcomes. As a result, this analysis provides clear, evidence-based guidance for designing personalized, step-by-step CR programs.

Despite these strengths, several limitations must be noted. First, the interactive nature of digital tools makes it impossible to blind participants and researchers, which may introduce bias into the results. Second, most of the supporting data comes from middle-income countries. While this highlights the success of group-based interventions in these regions, it makes it difficult to apply the findings globally—especially in high-income countries where standard clinical care is already highly advanced. Nevertheless, we confirmed the reliability of our findings through comprehensive quality checks, rigorous statistical testing, and a thorough literature search across multiple languages.

Furthermore, our analysis relies on summarized study data rather than individual patient records, and the participants were predominantly older men. This limits our ability to account for individual differences, such as gender, specific cardiovascular risks, and digital literacy. Therefore, caution is needed when applying these conclusions to other high-risk demographic groups. Finally, because the original trials reported adverse events differently, our safety analysis was limited to major cardiovascular events, preventing a detailed review of minor complications. To address these gaps, future research should analyze data at the individual patient level to allow for more precise and personalized treatment recommendations.

## Conclusions

This network meta-analysis of 44 randomized controlled trials, involving 8,495 patients with stable coronary heart disease, suggests that the clinical benefits of DHIs may be influenced by their delivery method, although the specific components of each intervention (e.g., psychological support, exercise modules) also play a substantial modifying role. Thus, strong conclusions about the inherent superiority of any single modality should be drawn cautiously. For improving QoL, telephone follow-up was significantly more effective than usual care and other digital approaches. Meanwhile, web-based platforms and VR were the most effective tools for reducing medium- to long-term anxiety and depression. Social media interventions also emerged as a versatile option with benefits across all three outcomes, though their modest effect sizes warrant further optimization of engagement strategies. This suggests that highly interactive digital environments are key to supporting mental health alongside physical recovery. However, safety data reveal a critical warning: while DHIs are largely safe overall, one-way mobile SMS interventions significantly increase the risk of major cardiovascular events when compared to interactive systems like telephone calls, mobile apps, and social media.

These findings challenge the standard “one-size-fits-all” approach to digital CR. Instead, they support a tailored strategy that matches specific digital tools to individual patient needs and recovery stages. Most importantly, one-way messaging systems can create a false sense of security or alarm fatigue, which may dangerously delay emergency care during acute heart events. Therefore, future clinical platforms must include two-way monitoring with early warning systems to ensure patient safety. Although limited by the open-label design of the original studies and a lack of individual patient data (IPD), this research provides strong guidance for deploying digital therapies in middle-income settings, where the incremental benefit over existing care appears most pronounced. For high-income countries, however, the evidence remains insufficient to recommend widespread adoption of single-modality DHIs without further context-specific validation. Large, multicentre studies using IPD are urgently needed to further refine this approach.

## Data Availability

The raw data supporting the conclusions of this article will be made available by the authors, without undue reservation.
