# Supplementary material for: Comparative efficacy of digital health intervention delivery modalities on quality of life, anxiety, and depression in coronary heart disease: a systematic review and network meta-analysis
Source: Front Psychiatry. 2026 Sep 28;17:1912762. doi: 10.3389/fpsyt.2026.1912762 (PMC13655782; doi:10.3389/fpsyt.2026.1912762)
Supplement: Supplementary file 1 [file SupplementaryFile1.docx]

**Supplementary Appendix**

**Comparative Efficacy of Digital Health Intervention Delivery Modalities on Quality of Life, Anxiety, and Depression in Coronary Heart Disease: A Systematic Review and Network Meta-Analysis**

Yiran Zhang, Tianyou Yu, Mutong Li, Lin Tian, Jiangbo Wang, Zhaofeng Li(待确定)

**Table of contents**

*[Appendix 1: Search strategy	2](#_Toc32198)*

*[Appendix 2: Characteristics of included studies	10](#_Toc12396)*

*[Appendix 3: Risk of bias of randomized clinical trials	20](#_Toc15479)*

*[Appendix 4: Evaluation of inconsistency and heterogeneity	23](#_Toc9939)*

*[Appendix 5: Network maps and forest plots of all outcomes	26](#_Toc3321)*

*[Appendix 6: SUCRA and cumulative probability plots	30](#_Toc14535)*

*[Appendix 7:Summary Ranking Table of the Effects of Digital Health Interventions.	34](#_Toc28879)*

*[Appendix 8: CINeMA Assessment	36](#_Toc21461)*

*[Appendix 9: Funnel plots	53](#_Toc4376)*

*[Appendix 10: Sensitivity analyses	57](#_Toc20287)*

*[Appendix 11: Subgroup analyses based on different intervention contents	58](#_Toc30762)*

*[Appendix 12:Impact of intervention duration on outcome measures	63](#_Toc17332)*

*[Appendix13:Comparison of the effectiveness of intervention measures across countries with different income levels	67](#_Toc31971)*

Appendix 1: Search strategy

**Table S1. Search strategy of PubMed**

| **#** | **Searches** |
| --- | --- |
| **1** | ((((("Coronary Disease"[Mesh Terms] OR "Coronary Artery Disease"[Mesh Terms] OR "Myocardial Ischemia"[Mesh Terms] OR "Acute Coronary Syndrome"[Mesh Terms] OR "Coronary Artery Bypass"[Mesh Terms] OR "Myocardial Revascularization"[Mesh Terms] OR "Percutaneous Coronary Intervention"[Mesh Terms]) |
| **2** | (((((((((((((((((((((((((((((((((((((Coronary Diseases[Title/Abstract] OR "Disease*, Coronary"[Title/Abstract] OR "Coronary Heart Disease*"[Title/Abstract] OR "Disease*, Coronary Heart"[Title/Abstract] OR "Heart Disease*, Coronary"[Title/Abstract] OR "Ischemia*, Myocardial"[Title/Abstract] OR "Myocardial Ischemia*"[Title/Abstract] OR "Ischemic Heart Disease*"[Title/Abstract] OR "Heart Disease*, Ischemic"[Title/Abstract] OR "Disease*, Ischemic Heart"[Title/Abstract] OR "Artery Disease*, Coronary"[Title/Abstract] OR "Coronary Artery Disease*"[Title/Abstract] OR "Left Main Coronary Artery Disease"[Title/Abstract] OR "Left Main Disease*"[Title/Abstract] OR "Left Main Coronary Disease"[Title/Abstract] OR "Coronary Arterioscleros*"[Title/Abstract] OR "Coronary Atheroscleros*"[Title/Abstract] OR "Acute Coronary Syndrome*"[Title/Abstract] OR "Coronary Syndrome*, Acute"[Title/Abstract] OR "Syndrome*, Acute Coronary"[Title/Abstract] OR "Coronary Intervention*, Percutaneous"[Title/Abstract] OR "Intervention*, Percutaneous Coronary"[Title/Abstract] OR "Percutaneous Coronary Interventions"[Title/Abstract] OR "Percutaneous Coronary Revascularization*"[Title/Abstract] OR "Coronary Revascularization*, Percutaneous"[Title/Abstract] OR "Revascularization*, Percutaneous Coronary"[Title/Abstract] OR "Percutaneous Transluminal Coronary Angioplast*"[Title/Abstract] OR "Artery Bypass*, Coronary"[Title/Abstract] OR "Bypass*, Coronary Artery"[Title/Abstract] OR "Coronary Artery Bypasses"[Title/Abstract] OR "Coronary Artery Bypass Surgery"[Title/Abstract] OR "Coronary Artery Bypass Grafting"[Title/Abstract] OR "Aortocoronary Bypass*"[Title/Abstract] OR "Bypass*, Aortocoronary"[Title/Abstract] OR "Bypass Surgery, Coronary Artery"[Title/Abstract] OR "Myocardial Revascularizations"[Title/Abstract] OR "Revascularization*, Myocardial"[Title/Abstract] OR "Internal Mammary Artery Implantation"[Title/Abstract]) |

| **3** | #1 OR #2 |
| --- | --- |
| **4** | (((((((((((((((((((((((((((((((("e advice"[Title/Abstract] OR "e-advice"[Title/Abstract] OR electronic*[Title/Abstract] OR "e learning"[Title/Abstract] OR "e-learning"[Title/Abstract] OR ehealth[Title/Abstract] OR "e health"[Title/Abstract] OR "e-health"[Title/Abstract] OR mhealth[Title/Abstract] OR "m health"[Title/Abstract] OR "m-health"[Title/Abstract] OR mcare[Title/Abstract] OR "m care"[Title/Abstract] OR "mobile communication"[Title/Abstract] OR technolog*[Title/Abstract] OR telecare[Title/Abstract] OR "tele-care"[Title/Abstract] OR telecommunication[Title/Abstract] OR "tele-communication"[Title/Abstract] OR telehealth[Title/Abstract] OR "tele-health"[Title/Abstract] OR telehomecare[Title/Abstract] OR "tele-homecare"[Title/Abstract] OR telemedicine[Title/Abstract] OR "tele-medicine"[Title/Abstract] OR telemanagement[Title/Abstract] OR "tele-management"[Title/Abstract] OR telemonitor*[Title/Abstract] OR "tele-monitor*"[Title/Abstract] OR telenursing[Title/Abstract] OR "tele-nursing"[Title/Abstract] OR telerehabilitation[Title/Abstract] OR "tele-rehabilitation"[Title/Abstract]) |
| **5** | ((((((((((((((((((((((((((((((((((((((((((((((((((((((((((internet[Title/Abstract] OR web[Title/Abstract] OR "internet based"[Title/Abstract] OR "internet-based"[Title/Abstract] OR "web based"[Title/Abstract] OR "web-based"[Title/Abstract] OR online[Title/Abstract] OR network[Title/Abstract] OR "social media"[Title/Abstract] OR "social networking"[Title/Abstract] OR mobile[Title/Abstract] OR "mobile technology"[Title/Abstract] OR app[Title/Abstract] OR apps[Title/Abstract] OR applications[Title/Abstract] OR blog[Title/Abstract] OR blogging[Title/Abstract] OR computer[Title/Abstract] OR email*[Title/Abstract] OR "e-mail"[Title/Abstract] OR "mobile device"[Title/Abstract] OR "mobile app"[Title/Abstract] OR "mobile application"[Title/Abstract] OR phone[Title/Abstract] OR telephone[Title/Abstract] OR "mobile phone"[Title/Abstract] OR "smart phone"[Title/Abstract] OR "cell phone"[Title/Abstract] OR "celluar phone"[Title/Abstract] OR "text messag*"[Title/Abstract] OR texting[Title/Abstract] OR "short messag*"[Title/Abstract] OR "short message service"[Title/Abstract] OR SMS[Title/Abstract] OR "instant messag*"[Title/Abstract] OR software[Title/Abstract] OR tablet[Title/Abstract] OR "tablet computer"[Title/Abstract] OR video[Title/Abstract] OR "video recording"[Title/Abstract] OR videoconferenc*[Title/Abstract] OR "video conferenc*"[Title/Abstract] OR "video-conference"[Title/Abstract] OR "video meeting"[Title/Abstract] OR "interactive video"[Title/Abstract] OR "TV meeting"[Title/Abstract] OR Wechat[Title/Abstract] OR webcast[Title/Abstract] OR ipad[Title/Abstract] OR teleconferenc*[Title/Abstract] OR "tele conferenc*"[Title/Abstract] OR virtual[Title/Abstract] OR "virtual realit*"[Title/Abstract] OR "virtual gam*"[Title/Abstract] OR VR[Title/Abstract] OR distant[Title/Abstract] OR wireless[Title/Abstract] OR remote[Title/Abstract] OR "remote consult*"[Title/Abstract]) |
| **6** | #4 OR #5 |
| **7** | ((("randomized controlled trial"[Title/Abstract] OR Randomized[Title/Abstract] OR RCT[Title/Abstract] OR "controlled clinical trial"[Title/Abstract]) |
| **8** | #3 AND #6 AND #7 |
| **9** | (((((((("Anxiety"[MeSH Terms] OR Angst[Title/Abstract] OR Nervousness[Title/Abstract] OR Hypervigilance[Title/Abstract] OR "Social Anxiety"[Title/Abstract] OR "Anxieties, Social"[Title/Abstract] OR "Anxiety, Social"[Title/Abstract] OR "Social Anxieties"[Title/Abstract] OR Anxiousness[Title/Abstract]) |
| **10** | ((((("Depression"[MeSH Terms] OR "Depressive Symptoms"[Title/Abstract] OR "Depressive Symptom"[Title/Abstract] OR "Symptom, Depressive"[Title/Abstract] OR "Emotional Depression"[Title/Abstract] OR "Depression, Emotional"[Title/Abstract]) |
| **11** | (((((((((("quality of life"[Title/Abstract] OR "life quality"[Title/Abstract] OR "life qualities"[Title/Abstract] OR "health-related quality of life"[Title/Abstract] OR "health related quality of life"[Title/Abstract] OR QOL[Title/Abstract] OR QoL[Title/Abstract] OR HRQL[Title/Abstract] OR HRQOL[Title/Abstract] OR HRQoL[Title/Abstract] OR "quality of living"[Title/Abstract]) |
| **12** | #9 OR #10 OR #11 |
| **13** | #8 AND #12 |

**Table S2. Search strategy of Embase**

| **#** | **Searches** |
| --- | --- |
| **1** | 'coronary artery disease'/exp OR 'ischemic heart disease'/exp OR 'acute coronary syndrome'/exp OR 'coronary artery bypass'/exp OR 'percutaneous coronary intervention'/exp OR 'coronary disease':ab,ti OR 'coronary artery disease':ab,ti OR 'myocardial ischemia':ab,ti OR 'acute coronary syndrome':ab,ti OR 'coronary artery bypass':ab,ti OR 'myocardial revascularization':ab,ti OR 'percutaneous coronary intervention':ab,ti OR 'coronary diseases':ab,ti OR 'disease*, coronary':ab,ti OR 'coronary heart disease*':ab,ti OR 'disease*, coronary heart':ab,ti OR 'heart disease*, coronary':ab,ti OR 'ischemia*, myocardial':ab,ti OR 'myocardial ischemia*':ab,ti OR 'ischemic heart disease*':ab,ti OR 'heart disease*, ischemic':ab,ti OR 'disease*, ischemic heart':ab,ti OR 'artery disease*, coronary':ab,ti OR 'coronary artery disease*':ab,ti OR 'left main coronary artery disease':ab,ti OR 'left main disease*':ab,ti OR 'left main coronary disease':ab,ti OR 'coronary arterioscleros*':ab,ti OR 'coronary atheroscleros*':ab,ti OR 'acute coronary syndrome*':ab,ti OR 'coronary syndrome*, acute':ab,ti OR 'syndrome*, acute coronary':ab,ti OR 'coronary intervention*, percutaneous':ab,ti OR 'intervention*, percutaneous coronary':ab,ti OR 'percutaneous coronary interventions':ab,ti OR 'percutaneous coronary revascularization*':ab,ti OR 'coronary revascularization*, percutaneous':ab,ti OR 'revascularization*, percutaneous coronary':ab,ti OR 'percutaneous transluminal coronary angioplast*':ab,ti OR 'artery bypass*, coronary':ab,ti OR 'bypass*, coronary artery':ab,ti OR 'coronary artery bypasses':ab,ti OR 'coronary artery bypass surgery':ab,ti OR 'coronary artery bypass grafting':ab,ti OR 'aortocoronary bypass*':ab,ti OR 'bypass*, aortocoronary':ab,ti OR 'bypass surgery, coronary artery':ab,ti OR 'myocardial revascularizations':ab,ti OR 'revascularization*, myocardial':ab,ti OR 'internal mammary artery implantation':ab,ti |
| **2** | 'telehealth'/exp OR 'telemedicine'/exp OR 'e advice':ab,ti OR 'e-advice':ab,ti OR 'electronic*':ab,ti OR 'e learning':ab,ti OR 'ehealth':ab,ti OR 'e health':ab,ti OR 'e-health':ab,ti OR 'mhealth':ab,ti OR 'm health':ab,ti OR 'm-health':ab,ti OR 'mcare':ab,ti OR 'm care':ab,ti OR 'mobile communication':ab,ti OR 'technolog*':ab,ti OR 'telecare':ab,ti OR 'tele-care':ab,ti OR 'telecommunication':ab,ti OR 'tele-communication':ab,ti OR 'telehealth':ab,ti OR 'tele-health':ab,ti OR 'telehomecare':ab,ti OR 'tele-homecare':ab,ti OR 'telemedicine':ab,ti OR 'tele-medicine':ab,ti OR 'telemanagement':ab,ti OR 'tele-management':ab,ti OR 'telemonitor*':ab,ti OR 'tele-monitor*':ab,ti OR 'telenursing':ab,ti OR 'tele-nursing':ab,ti OR 'telerehabilitation':ab,ti OR 'tele-rehabilitation':ab,ti |
| **3** | 'social media'/exp OR 'mobile application'/exp OR 'internet':ab,ti OR 'web':ab,ti OR 'internet based':ab,ti OR 'internet-based':ab,ti OR 'web based':ab,ti OR 'web-based':ab,ti OR 'online':ab,ti OR 'network':ab,ti OR 'social media':ab,ti OR 'social networking':ab,ti OR 'mobile':ab,ti OR 'mobile technology':ab,ti OR 'app':ab,ti OR 'apps':ab,ti OR 'applications':ab,ti OR 'blog':ab,ti OR 'blogging':ab,ti OR 'computer':ab,ti OR 'email*':ab,ti OR 'e-mail':ab,ti OR 'mobile device':ab,ti OR 'mobile app':ab,ti OR 'mobile application':ab,ti OR 'phone':ab,ti OR 'text messag*':ab,ti OR 'texting':ab,ti OR 'short messag*':ab,ti OR 'short message service':ab,ti OR 'SMS':ab,ti OR 'instant messag*':ab,ti OR 'software':ab,ti OR 'tablet':ab,ti OR 'tablet computer':ab,ti OR 'video':ab,ti OR 'video recording':ab,ti OR 'videoconferenc*':ab,ti OR 'video conferenc*':ab,ti OR 'video-conference':ab,ti OR 'video meeting':ab,ti OR 'interactive video':ab,ti OR 'TV meeting':ab,ti OR 'Wechat':ab,ti OR 'webcast':ab,ti OR 'ipad':ab,ti OR 'teleconferenc*':ab,ti OR 'tele conferenc*':ab,ti OR 'virtual':ab,ti OR 'virtual realit*':ab,ti OR 'virtual gam*':ab,ti OR 'VR':ab,ti OR 'distant':ab,ti OR 'wireless':ab,ti OR 'remote':ab,ti OR 'remote consult':ab,ti |
| **4** | 'randomized controlled trial'/exp OR 'randomized controlled trial':ab,ti OR 'randomized':ab,ti OR 'rct':ab,ti OR 'controlled clinical trial':ab,ti |
| **5** | 'anxiety'/exp OR 'depression'/exp OR 'anxiety':ab,ti OR 'angst':ab,ti OR 'nervousness':ab,ti OR 'hypervigilance':ab,ti OR 'social anxiety':ab,ti OR 'anxieties, social':ab,ti OR 'anxiety, social':ab,ti OR 'social anxieties':ab,ti OR 'anxiousness':ab,ti OR 'depression':ab,ti OR 'depressive symptoms':ab,ti OR 'depressive symptom':ab,ti OR 'symptom, depressive':ab,ti OR 'emotional depression':ab,ti OR 'depression, emotional':ab,ti |
| **6** | 'quality of life'/exp OR 'quality of life':ab,ti OR 'life quality':ab,ti OR 'life qualities':ab,ti OR 'health-related quality of life':ab,ti OR 'health related quality of life':ab,ti OR 'qol':ab,ti OR 'qol':ab,ti OR 'hrql':ab,ti OR 'hrqol':ab,ti OR 'hrqol':ab,ti OR 'quality of living':ab,ti |
| **7** | #2 OR #3 |
| **8** | #5 OR #6 |
| **9** | #1 AND #4 AND #7 AND #8 |

**Table S3. Search strategy of Web of Science**

| **#** | **Searches** |
| --- | --- |
| **1** | TS=("Coronary Disease" OR "Coronary Artery Disease" OR "Myocardial Ischemia" OR "Acute Coronary Syndrome" OR "Coronary Artery Bypass" OR "Myocardial Revascularization" OR "Percutaneous Coronary Intervention" OR "Coronary Diseases" OR "Disease*, Coronary" OR "Coronary Heart Disease*" OR "Disease*, Coronary Heart" OR "Heart Disease*, Coronary" OR "Ischemia*, Myocardial" OR "Myocardial Ischemia*" OR "Ischemic Heart Disease*" OR "Heart Disease*, Ischemic" OR "Disease*, Ischemic Heart" OR "Artery Disease*, Coronary" OR "Coronary Artery Disease*" OR "Left Main Coronary Artery Disease" OR "Left Main Disease*" OR "Left Main Coronary Disease" OR "Coronary Arterioscleros*" OR "Coronary Atheroscleros*" OR "Acute Coronary Syndrome*" OR "Coronary Syndrome*, Acute" OR "Syndrome*, Acute Coronary" OR "Coronary Intervention*, Percutaneous" OR "Intervention*, Percutaneous Coronary" OR "Percutaneous Coronary Interventions" OR "Percutaneous Coronary Revascularization*" OR "Coronary Revascularization*, Percutaneous" OR "Revascularization*, Percutaneous Coronary" OR "Percutaneous Transluminal Coronary Angioplast*" OR "Artery Bypass*, Coronary" OR "Bypass*, Coronary Artery" OR "Coronary Artery Bypasses" OR "Coronary Artery Bypass Surgery" OR "Coronary Artery Bypass Grafting" OR "Aortocoronary Bypass*" OR "Bypass*, Aortocoronary" OR "Bypass Surgery, Coronary Artery" OR "Myocardial Revascularizations" OR "Revascularization*, Myocardial" OR "Internal Mammary Artery Implantation") |
| **2** | TS=("e advice" OR "e-advice" OR "electronic*" OR "e learning" OR "e-learning" OR "ehealth" OR "e health" OR "e-health" OR "mhealth" OR "m health" OR "m-health" OR "mcare" OR "m care" OR "mobile communication" OR "technolog*" OR "telecare" OR "tele-care" OR "telecommunication" OR "tele-communication" OR "telehealth" OR "tele-health" OR "telehomecare" OR "tele-homecare" OR "telemedicine" OR "tele-medicine" OR "telemanagement" OR "tele-management" OR "telemonitor*" OR "tele-monitor*" OR "telenursing" OR "tele-nursing" OR "telerehabilitation" OR "tele-rehabilitation" OR "internet" OR "web" OR "internet based" OR "internet-based" OR "web based" OR "web-based" OR "online" OR "network" OR "social media" OR "social networking" OR "mobile" OR "mobile technology" OR "app" OR "apps" OR "applications" OR "blog" OR "blogging" OR "computer" OR "email" OR "e-mail" OR "mobile device" OR "mobile app" OR "mobile application" OR "phone" OR "telephone" OR "mobile phone" OR "smart phone" OR "cell phone" OR "celluar phone" OR "text messag*" OR "texting" OR "short messag*" OR "short message service" OR "SMS" OR "instant messag*" OR "software" OR "tablet" OR "tablet computer" OR "video" OR "video recording" OR "videoconferenc*" OR "video conferenc*" OR "video-conference" OR "video meeting" OR "interactive video" OR "TV meeting" OR "Wechat" OR "webcast" OR "ipad" OR "teleconferenc*" OR "tele conferenc*" OR "virtual" OR "virtual realit*" OR "virtual gam*" OR "VR" OR "distant" OR "wireless" OR "remote" OR "remote consult*") |
| **3** | TS=("Anxiety" OR "Angst" OR "Nervousness" OR "Hypervigilance" OR "Social Anxiety" OR "Anxieties, Social" OR "Anxiety, Social" OR "Social Anities" OR "Anxiousness" OR "Depression" OR "Depressive Symptoms" OR "Depressive Symptom" OR "Symptom, Depressive" OR "Emotional Depression" OR "Depression, Emotional" OR "quality of life" OR "life quality" OR "life qualities" OR "health-related quality of life" OR "health related quality of life" OR "QOL" OR "QoL" OR "HRQL" OR "HRQOL" OR "HRQoL" OR "quality of living") |
| **4** | TS=("randomized controlled trial" OR "Randomized" OR "RCT" OR "controlled clinical trial") |
| **5** | #1 AND #2 AND #3 AND #4 |

**Table S4. Search strategy of Cochrane Central Register of Controlled Trials**

| **#** | **Searches** |
| --- | --- |
| **1** | ("Coronary Disease" OR "Coronary Artery Disease" OR "Myocardial Ischemia" OR "Acute Coronary Syndrome" OR "Coronary Artery Bypass" OR "Myocardial Revascularization" OR "Percutaneous Coronary Intervention" OR "Coronary Diseases" OR "Disease*, Coronary" OR "Coronary Heart Disease*" OR "Disease*, Coronary Heart" OR "Heart Disease*, Coronary" OR "Ischemia*, Myocardial" OR "Myocardial Ischemia*" OR "Ischemic Heart Disease*" OR "Heart Disease*, Ischemic" OR "Disease*, Ischemic Heart" OR "Artery Disease*, Coronary" OR "Coronary Artery Disease*" OR "Left Main Coronary Artery Disease" OR "Left Main Disease*" OR "Left Main Coronary Disease" OR "Coronary Arterioscleros*" OR "Coronary Atheroscleros*" OR "Acute Coronary Syndrome*" OR "Coronary Syndrome*, Acute" OR "Syndrome*, Acute Coronary" OR "Coronary Intervention*, Percutaneous" OR "Intervention*, Percutaneous Coronary" OR "Percutaneous Coronary Interventions" OR "Percutaneous Coronary Revascularization*" OR "Coronary Revascularization*, Percutaneous" OR "Revascularization*, Percutaneous Coronary" OR "Percutaneous Transluminal Coronary Angioplast*" OR "Artery Bypass*, Coronary" OR "Bypass*, Coronary Artery" OR "Coronary Artery Bypasses" OR "Coronary Artery Bypass Surgery" OR "Coronary Artery Bypass Grafting" OR "Aortocoronary Bypass*" OR "Bypass*, Aortocoronary" OR "Bypass Surgery, Coronary Artery" OR "Myocardial Revascularizations" OR "Revascularization*, Myocardial" OR "Internal Mammary Artery Implantation"):ti,ab,kw |
| **2** | ("e advice" OR "e-advice" OR electronic* OR "e learning" OR "e-learning" OR ehealth OR "e health" OR "e-health" OR mhealth OR "m health" OR "m-health" OR mcare OR "m care" OR "mobile communication" OR technolog* OR telecare OR "tele-care" OR telecommunication OR "tele-communication" OR telehealth OR "tele-health" OR telehomecare OR "tele-homecare" OR telemedicine OR "tele-medicine" OR telemanagement OR "tele-management" OR telemonitor* OR "tele-monitor*" OR telenursing OR "tele-nursing" OR telerehabilitation OR "tele-rehabilitation" OR internet OR web OR "internet based" OR "internet-based" OR "web based" OR "web-based" OR online OR network OR "social media" OR "social networking" OR mobile OR "mobile technology" OR app OR apps OR applications OR blog OR blogging OR computer OR email OR "e-mail" OR "mobile device" OR "mobile app" OR "mobile application" OR phone OR telephone OR "mobile phone" OR "smart phone" OR "cell phone" OR "celluar phone" OR "text messag*" OR texting OR "short messag*" OR "short message service" OR SMS OR "instant messag*" OR software OR tablet OR "tablet computer" OR video OR "video recording" OR videoconferenc* OR "video conferenc*" OR "video-conference" OR "video meeting" OR "interactive video" OR "TV meeting" OR Wechat OR webcast OR ipad OR teleconferenc* OR "tele conferenc*" OR virtual OR "virtual realit*" OR "virtual gam*" OR VR OR distant OR wireless OR remote OR "remote consult*"):ti,ab,kw |
| **3** | (Anxiety OR Angst OR Nervousness OR Hypervigilance OR "Social Anxiety" OR "Anxieties, Social" OR "Anxiety, Social" OR "Social Anxieties" OR Anxiousness OR Depression OR "Depressive Symptoms" OR "Depressive Symptom" OR "Symptom, Depressive" OR "Emotional Depression" OR "Depression, Emotional" OR "quality of life" OR "life quality" OR "life qualities" OR "health-related quality of life" OR "health related quality of life" OR QOL OR QoL OR HRQL OR HRQOL OR HRQoL OR "quality of living"):ti,ab,kw |
| **4** | ("randomized controlled trial" OR Randomized OR RCT OR "controlled clinical trial"):ti,ab,kw |
| **5** | #1 AND #2 AND #3 AND #4 |

**Table S5. Search strategy of CNKI**

| **#** | **Searches** |
| --- | --- |
| **1** | SU = '冠心病' + '冠状动脉粥样硬化性心脏病' + '心肌缺血' + '冠脉综合征' + '冠状动脉旁路' + '心肌血管重建术' + '经皮冠状动脉介入治疗' |
| **2** | SU = '电子健康' + '数字健康干预' + '应用程序' + '用户软件' + '应用软件' + '网站' + '电话' + '虚拟现实' + 'VR' |
| **3** | #1 AND #2 |

**Table S6. Search strategy of VIP**

| **#** | **Searches** |
| --- | --- |
| **1** | M=(冠心病 OR 冠状动脉粥样硬化性心脏病 OR 心肌缺血 OR 冠脉综合征 OR 冠状动脉旁路 OR 心肌血管重建术 OR 经皮冠状动脉介入治疗) |
| **2** | M=(电子健康 OR 数字健康干预 OR 应用程序 OR 用户软件 OR 应用软件 OR 网站 OR 电话 OR 虚拟现实 OR VR) |
| **3** | #1 AND #2 |

**Table S7. Search strategy of Wanfang Data**

| **#** | **Searches** |
| --- | --- |
| **1** | (题名:"冠心病" OR 关键词:"冠心病" OR 题名:"冠状动脉粥样硬化性心脏病" OR 关键词:"冠状动脉粥样硬化性心脏病" OR 题名:"心肌缺血" OR 关键词:"心肌缺血" OR 题名:"冠脉综合征" OR 关键词:"冠脉综合征" OR 题名:"冠状动脉旁路" OR 关键词:"冠状动脉旁路" OR 题名:"心肌血管重建术" OR 关键词:"心肌血管重建术" OR 题名:"经皮冠状动脉介入治疗" OR 关键词:"经皮冠状动脉介入治疗") |
| **2** | (题名:"电子健康" OR 关键词:"电子健康" OR 题名:"数字健康干预" OR 关键词:"数字健康干预" OR 题名:"应用程序" OR 关键词:"应用程序" OR 题名:"用户软件" OR 关键词:"用户软件" OR 题名:"应用软件" OR 关键词:"应用软件" OR 题名:"网站" OR 关键词:"网站" OR 题名:"电话" OR 关键词:"电话" OR 题名:"虚拟现实" OR 关键词:"虚拟现实" OR 题名:"VR" OR 关键词:"VR") |
| **3** | #1 AND #2 |

**Table S8. Search strategy of SinoMed**

| **#** | **Searches** |
| --- | --- |
| **1** | ("冠心病"[常用字段:智能] OR "冠状动脉粥样硬化性心脏病"[常用字段:智能] OR "心肌缺血"[常用字段:智能] OR "冠脉综合征"[常用字段:智能] OR "冠状动脉旁路"[常用字段:智能] OR "心肌血管重建术"[常用字段:智能] OR "经皮冠状动脉介入治疗"[常用字段:智能]) |
| **2** | ("电子健康"[常用字段:智能] OR "数字健康干预"[常用字段:智能] OR "应用程序"[常用字段:智能] OR "用户软件"[常用字段:智能] OR "应用软件"[常用字段:智能] OR "网站"[常用字段:智能] OR "电话"[常用字段:智能] OR "虚拟现实"[常用字段:智能] OR "VR"[常用字段:智能]) |
| **3** | #1 AND #2 |

Appendix 2: Characteristics of included studies

**Table S2.1:** Baseline of characteristics of included studies

| **Study** | **Country and region** | **Design** | **Intervention Duration (months)** | **Number of participants** | **Retention rate** | **Intervention & Control** | **Age (mean ± SD), years** | **Male %** | **Participant Characteristics** | **Outcomes** | **Scales** |
| --- | --- | --- | --- | --- | --- | --- | --- | --- | --- | --- | --- |
|  |  |  |  |  |  |  |  |  |  |  |  |
| Reid 2011 | Canada | RCT | 6 | 223 | 65.20% | Web-based Platform | 56.7 ± 9.0 | 82.60% | Clinically stable post-PCI ACS patients in the pre-discharge phase, transitioning to outpatient/home-based cardiac rehabilitation. | A | MacNew |
|  |  |  |  |  | 73.10% | Usual care | 56.0 ± 9.0 | 86.10% |  |  |  |
| Dorje 2019 | China | RCT | 6 | 312 | 85.90% | Social Media | 59.1 ± 9.4 | 82.00% | Hemodynamically stable post-PCI CHD patients past the acute phase, eligible for early cardiac rehabilitation. | ABC | SF-12、GAD-7、PHQ-9 |
|  |  |  |  |  | 84.00% | Usual care | 61.9 ± 8.7 | 81.00% |  |  |  |
| Snoek 2019 | Netherlands | RCT | 6 | 122 | 96.70% | Wearables/Monitoring | 60.0 ± 8.4 | 82.00% | CAD patients post-Phase II CR; home-based Phase III. | ABC | MacNew、HADS、PHQ-9 |
|  |  |  |  |  | 96.70% | Usual care | 59.0 ± 10.7 | 82.00% |  |  |  |
| Chow 2022 | Australia | RCT | 12 | 1424 | 97.30% | Mobile SMS | 58.0 ± 10.4 | 79.50% | ACS patients (AMI or ischemic hospitalization); post-acute phase. | ABC | SF-12、GAD-7、PHQ-9 |
|  |  |  |  |  | 96.30% | Usual care | 58.0 ± 10.9 | 79.00% |  |  |  |
| Vieira 2017 | Portugal | RCT | 6 | 46 | 73.30% | VR | 55 ± 9.0 | 100.00% | Stable CHD patients who completed Phase II CR; Phase III. | ABC | MacNew、DASS-21 |
|  |  |  |  |  | 68.80% | Usual care | 59 ± 5.8 | 100.00% |  |  |  |
| Cheung 2023 | Australia | RCT | 6 | 506 | 89.10% | Mobile SMS | 61.0±11.1 | 71.60% | Adults with T2DM, CHD | AC | SF-12、PHQ-9 |
|  |  |  |  |  | 90.00% | Usual care | 61.9±12.0 | 71.40% |  |  |  |
| Pitta 2022 | Brazil | RCT | 3 | 110 | 98.10% | Telephone Follow-up | 66.9±10.4 | 66.70% | Post-initial PCI. | BC | HADS |
|  |  |  |  |  | 100% | Usual care | 61.1±9.5 | 62.50% |  |  |  |
| Islam 2019 | Australia | RCT | 6 | 710 | 94.60% | Mobile SMS | 57.9±9.0 | 81.70% | Confirmed CHD (including history of MI, CABG, PCI, or angiographically documented CHD). | C | PHQ-9 |
|  |  |  |  |  | 97.80% | Usual care | 57.3±9.2 | 82.60% |  |  |  |
| Hisam 2022 | Pakistan | RCT | 6 | 160 | 87.50% | Mobile App | 53.70±9.56 | 63.75% | Post-ACS (including STEMI, NSTEMI, and unstable angina). | A | MacNew、SF-12 |
|  |  |  |  |  | 61.25% | Usual care | 51.64±7.13 | 93.75% |  |  |  |
| Bernal-Jiménez 2024 | Spain | RCT | 9 | 134 | 100% | Mobile App | 57.70±8.16 | 79.10% | CAD patients post-PCI. | A | SF-12 |
|  |  |  |  |  | 91% | Usual care | 61.46±9.47 | 63.90% |  |  |  |
| Su 2021 | China | RCT | 3 | 146 | 90.40% | Social Media | 55.53±7.30 | 82.20% | Discharged adults hospitalized for initial CHD diagnosis or exacerbation of pre-existing CHD (including PCI or medical therapy only). | ABC | MacNew、DASS-21 |
|  |  |  |  |  | 79.50% | Usual care | 56.03±7.02 | 84.90% |  |  |  |
| Kumar 2024 | India | RCT | 3 | 40 | 100% | Social Media | 57.75±4.84 | 90.00% | Low-risk patients (aged 35–65 years) post-conventional CABG referred for Phase II cardiac rehabilitation. | A | WHOQOL |
|  |  |  |  |  | 100% | Usual care | 55.94±6.91 | 90.00% |  |  |  |
| Avila 2018 | Belgium | RCT | 3 | 90 | 93.30% | Wearables/Monitoring | 58.6±13.0 | 86.70% | CAD patients post-completion of outpatient CR. | A | SF-36 |
|  |  |  |  |  | 86.70% | Usual care | 61.7±7.7 | 90.00% |  |  |  |
|  |  |  |  |  | 100% | Cardiac rehabilitation | 61.9 ± 7.3 | 90.00% |  |  |  |
| Fu 2025 | China | RCT | 6 | 60 | 100% | Web-based Platform | 73.33±5.70 | 56.70% | Low-to-moderate risk patients post-emergency PCI with stable vital signs, recovering well and cleared for regular rehabilitation. | ABC | SF-36、SAS、SDS |
|  |  |  |  |  | 100% | Usual care | 72.47±5.50 | 53.30% |  |  |  |
| Bretschneider 2024 | Germany | RCT | 1—3 | 354 | 78.90% | Mobile App | 50.23±5.34 | 75.80% | CHD patients with prior participation in cardiac rehabilitation (CR). | A | HeartQol |
|  |  |  |  |  | 61.60% | Usual care | 51.16±4.31 | 100.00% |  |  |  |
| Li 2024 | China | RCT | 12 | 174 | 93.10% | Social Media | 61.09±8.08 | 55.20% | CAD patients post-CABG surgery. | ABC | SF-12、HADS |
|  |  |  |  |  | 89.70% | Usual care | 60.80±9.39 | 58.60% |  |  |  |
| Hua 2024 | China | RCT | 1 | 92 | 91.30% | Telephone Follow-up | 60.50±8.90 | 80.95% | Post-PCI with stenting. | A | SF-36 |
|  |  |  |  |  | 93.50% | Usual care | 60.86±8.85 | 81.40% |  |  |  |
| Pfaeffli Dale 2015 | New Zealand | RCT | 6 | 123 | 93.40% | Mobile SMS | 59.0±10.5 | 79.00% | Adults with CHD, including recent MI, angina, or history of revascularization (PCI/CABG). | BC | HADS |
|  |  |  |  |  | 95.20% | Cardiac rehabilitation | 59.9±11.8 | 84.00% |  |  |  |
| Gu 2023 | China | RCT | 3 | 180 | 93.30% | Social Media | 62.8±6.5 | 73.30% | Post-PCI for ACS. | A | EQ-VAS |
|  |  |  |  |  | 90.00% | Usual care | 64.0±5.8 | 72.20% |  |  |  |
| Ma 2021 | China | RCT | 12 | 140 | 94.30% | Social Media | 65.2±7.3 | 82.90% | Post-CABG for unprotected left main coronary artery disease (ULMCAD). | ABC | SF-12、HADS |
|  |  |  |  |  | 91.40% | Usual care | 65.1±7.5 | 75.70% |  |  |  |
| Zou 2024 | China | RCT | 1.5 | 178 | 85.40% | Social Media | 59.48±8.95 | 66.30% | ACS patients (including unstable angina and acute myocardial infarction). | BC | GAD-7、PHQ-9 |
|  |  |  |  |  | 78.70% | Usual care | 58.02±8.90 | 70.80% |  |  |  |
| Zhang 2024 | China | RCT | 24 | 552 | NA | Social Media | 59.33±9.69 | 68.70% | Adult CHD patients with stable physiological parameters, undergoing pre-discharge evaluation to bridge into outpatient digital cardiac rehabilitation. | ABC | EQ-VAS、EQ-5D_5L、GAD-7、PHQ-9 |
|  |  |  |  |  | NA | Usual care | 62.33±10.43 | 68.70% |  |  |  |
| Ellis 2025 | Australia | RCT | 3 | 84 | 71.40% | Mobile App | 59 ± 10 | 86.00% | Low-to-moderate risk ACS patients prior to discharge, preparing to initiate Phase II/III cardiac rehabilitation. | A | EQ-VAS、EQ-5D_5L |
|  |  |  |  |  | 71.40% | Usual care | 60 ± 12 | 86.00% |  |  |  |
| Wei 2023 | China | RCT | 3 | 80 | 100% | Social Media | NA | 67.50% | Adult CHD patients post-successful PCI in the home-based cardiac rehabilitation phase. | A | CQQC |
|  |  |  |  |  | 100% | Usual care | NA | 85.00% |  |  |  |
| Hong 2021 | Taiwan, China | RCT | 3 | 60 | NA | Wearables/Monitoring | 60.10 ± 9.06 | 70.00% | Adults with established CAD (MI, CABG, or PCI); home-based secondary prevention and CR. | A | WHOQOL |
|  |  |  |  |  | NA | Usual care | 59.97 ± 8.16 | 73.30% |  |  |  |
| Zhang 2020 | China | RCT | 6 | 88 | 100.00% | Mobile App | 60.5±6.2 | 63.60% | CHD patients following their first successful PCI. | BC | SAS、SDS |
|  |  |  |  |  | 100.00% | Usual care | 59.8±5.8 | 59.10% |  |  |  |
| An 2015 | China | RCT | 3 | 230 | 99% | Telephone Follow-up | 53.0±13.5 | 56.50% | CAD patients post-successful PCI, excluding those with heart failure or other severe comorbidities. | A | SF-36 |
|  |  |  |  |  | 99% | Usual care | 56.5±14.5 | 53.00% |  |  |  |
| Chen 2017 | China | RCT | 6 | 150 | 98% | Telephone Follow-up | 69.49 ± 4.83 | 53.30% | Stable discharged CHD; on standard medical therapy; excluding severe HF/critical illness. | A | MLHFQ |
|  |  |  |  |  | 98% | Usual care | 70.58 ± 3.79 | 66.70% |  |  |  |
| Dalli Peydró 2022 | Spain | RCT | 10 | 67 | 93% | Wearables/Monitoring | 57.5±9.0 | 87.10% | Low-risk ACS patients post-discharge with preserved ejection fraction. | ABC | EQ-VAS、EQ-5D_5L、HADS |
|  |  |  |  |  | 82% | Cardiac rehabilitation | 54.7±9.9 | 89.30% |  |  |  |
| Kraal 2017 | Netherlands | RCT | 3 | 90 | 82.20% | Wearables/Monitoring | 60.5±8.8 | 88.90% | Post-ACS/PCI/CABG; cardiac rehab phase; low-to-moderate risk. | ABC | MacNew、HADS |
|  |  |  |  |  | 91.10% | Cardiac rehabilitation | 57.7±8.7 | 88.90% |  |  |  |
| Zhang 2025 | China | RCT | 3 | 62 | 91.40% | Mobile App | 62.67±4.89 | 56.30% | Adults with established CHD (including post-PCI, post-MI, or stable angina), excluding those with NYHA Class III-IV heart failure. | ABC | SF-36、GAD-7、PHQ-9 |
|  |  |  |  |  | 85.70% | Usual care | 57.2±13.0 | 63.30% |  |  |  |
| Maddison 2019 | New Zealand | RCT | 3 | 162 | 82.90% | Wearables/Monitoring | 61.0±13.2 | 84.20% | Clinically stable adult patients with a recent record of coronary heart disease (CHD). | A | EQ-VAS |
|  |  |  |  |  | 90.00% | Cardiac rehabilitation | 61.5±12.2 | 87.50% |  |  |  |
| Liu 2026 | China | RCT | 3 | 180 | 90.00% | Social Media | 63.86±9.17 | 81.10% | Following percutaneous coronary intervention (PCI). | A | SF-36 |
|  |  |  |  |  | 87% | Cardiac rehabilitation | 62.68±8.58 | 74.40% |  |  |  |
| Krzowski 2023 | Poland | RCT | 6 | 100 | 74% | Mobile App | 56.8 ± 9.23 | 68.00% | Clinically stable AMI patients post-acute treatment, undergoing pre-discharge assessment before entering outpatient cardiac rehabilitation. | A | MacNew、EQ-VAS |
|  |  |  |  |  | 74% | Usual care | 63.42 ± 11.4 | 61.00% |  |  |  |
| Bai Yang 2016 | China | RCT | 12 | 150 | 96.00% | Mobile App | 57.12±11.01 | 59.70% | Stable CHD patients post-hospitalization; home-based cardiac rehabilitation phase. | A | GQOLI-74 |
|  |  |  |  |  | 92.00% | Usual care | 57.12±11.01 | 60.30% |  |  |  |
| Batalik 2021 | Czech | RCT | 3 | 56 | 82.10% | Wearables/Monitoring | 56.1±6.8 | 78.00% | CAD; post-PCI/CABG. | A | SF-36 |
|  |  |  |  |  | 75.00% | Cardiac rehabilitation | 57.1±7.9 | 86.00% |  |  |  |
| Liang Jing 2023 | China | RCT | 3 | 95 | 93.80% | Mobile App | 58.47±7.12 | 71.10% | Low-to-moderate risk CHD; excluding severe HF/recent MI; post-discharge exercise CR phase. | ABC | SF-36、GAD-7、PHQ-9 |
|  |  |  |  |  | 95.70% | Usual care | 59.62±7.01 | 71.10% |  |  |  |
| Jóźwik 2021 | Poland | RCT | 0.75 | 100 | 56.00% | VR | 66.00±9.73 | 39.30% | IHD patients; outpatient Phase II cardiac rehabilitation. | BC | HADS |
|  |  |  |  |  | 98.00% | Cardiac rehabilitation | 63.96±6.89 | 49.00% |  |  |  |
| Li 2025 | China | RCT | 6 | 294 | 87.80% | Mobile App | 64.76±7.79 | 83.70% | Community-dwelling patients with coronary artery disease (CAD). | A | MacNew |
|  |  |  |  |  | 86.40% | Usual care | 66.65±8.07 | 85.70% |  |  |  |
| Gong Xue 2025 | China | RCT | 3 | 92 | 100% | VR | 51.82±7.95 | 52.20% | Clinically stable post-PCI CHD patients participating in Phase II/III cardiac rehabilitation | BC | SAS、SDS |
|  |  |  |  |  | 100% | Usual care | 52.67±8.04 | 56.50% |  |  |  |
| Zheng 2024 | China | RCT | 3 | 110 | 96.40% | Wearables/Monitoring | 63.49±9.53 | 60.40% | CHD patients following their first PCI. | A | SF-12 |
|  |  |  |  |  | 96.40% | Usual care | 63.04±10.44 | 56.60% |  |  |  |
| Fang 2018 | China | RCT | 1.5 | 80 | 82.50% | Wearables/Monitoring | 60.24±9.35 | 63.60% | Low-risk patients post-PCI. | AC | SF-36、CDS |
|  |  |  |  |  | 85.00% | Usual care | 61.41±10.17 | 61.80% |  |  |  |
| García-Bravo 2020 | Spain | RCT | 2 | 26 | 76.90% | VR | 48.70±6.66 | 100.00% | Patients with ischemic heart disease (IHD); low-risk Phase II cardiac rehabilitation. | AC | SF-36、Beck-Ⅱ |
|  |  |  |  |  | 76.90% | Cardiac rehabilitation | 53.7±10.30 | 100.00% |  |  |  |
| Chong, 2025 | Malaysia | RCT | 3 | 160 | 81.30% | Wearables/Monitoring | 61.6±8.2 | 82.60% | CHD patients post-revascularization (PCI, CABG, or thrombolysis) with low-to-moderate risk. | BC | HADS |
|  |  |  |  |  | 63.80% | Cardiac rehabilitation | 60.2±9.0 | 87.00% |  |  |  |

**A**: health-related quality of life; **B**: anxiety; **C**:depression.

**SF-36**:Short Form Health Survey-36 ;**SF-12**: Short Form Health Survey-12;**WHOQOL**:World Health Organization Quality of Life;

**MacNew**:MacNew Heart Disease Health-Related Quality of Life; **GQOLI-74**:Generic Quality of Life Inventory-74; **HeartQoL**:Heart Quality of Life Questionnaire;

**MLHFQ**:Minnesota Living with Heart Failure Questionnaire; **CQQC**:China Questionnaire of Quality of Life in Patients with Cardiovascular Diseases;

**EQ- VAS**:EuroQol Visual Analogue Scale; **EQ-5D_5L**:EuroQol-5 Dimension, 5-Level; **GAD-7**:General Anxiety Disorder-7;

**HADS**:Hospital Anxiety and Depression Scale; **DASS-21**:Depression, Anxiety, and Stress Scale-21; **SAS**:Self-Rating Anxiety Scale; **PHQ-9**:Patient Health Questionnaire-9

**SDS**:Self-Rating Depression Scale; **Beck-Ⅱ**:Beck Depression Inventory-II; **CDS**:Cardiac Depression Scale

Note: See Table 2 in the main text for a note on the heterogeneity of the TEXTMEDS trial (Chow 2022).

**Table S2.2: Checklist for Digital Health Interventions and Comparators**

| **Intervention/Control Measures** | **Definition** | **Core Components** | **Interaction & Technical Traits** |
| --- | --- | --- | --- |
| Mobile SMS | Sending short text messages to patients' mobile phones via mobile communication networks. | Delivery of health education, medication reminders, and exercise check-in encouragement. | Minimal technical dependency. Typically involves automated triggers based on preset programs with a lack of real-time feedback; emphasizes behavioral nudging and the delivery of simple information. |
| Telephone Follow-up | Continuity of care services provided by healthcare professionals through voice calls. | Motivational interviewing, psychological support, symptom consultation, and guidance for overcoming barriers. | High interpersonal interactivity. Relies on traditional telecommunication technology; characterized by exceptional flexibility and emotional support, allowing for real-time adjustments to guidance strategies based on patient feedback. |
| Mobile App | Dedicated interactive software installed on smartphones/tablets (e.g., mebix, MCard). | Structured educational videos, exercise logs, avatar-guided navigation, and goal setting. | Highly integrated functionality. Utilizes smartphone sensors, push notifications, and algorithms to achieve semi-automated feedback; often incorporates avatars or gamification elements to enhance immersion. |
| Social Media | Patient management utilizing existing popular social media platforms (e.g., WeChat groups). | Establishing group-based Q&A sessions, pushing science communication articles/videos, and sharing patient experiences. | Dual synchronous and asynchronous characteristics. Leverages social connectivity to generate group accountability; information dissemination is fragmented and high-frequency, containing strong psychosocial support attributes. |
| Wearables/Monitoring | Remote collection and transmission of patients' physiological parameters using sensor devices. | Monitoring of blood pressure, heart rate, step count, and sleep, along with cloud-based data alerts. | Passive data collection. Achieves a "patient collection - system analysis - clinician alert" closed loop via Bluetooth or cloud technology; provides objective, quantified physiological feedback and requires high technical precision. |
| VR | Immersive intervention technology based on computer-generated 3D simulated environments. | Immersive aerobic exercise training, guided breathing relaxation exercises, and scenario-based rehabilitation. | Fully digital closed-loop environment. Facilitates mind-body integration exercises through multi-sensory visual and auditory feedback; possesses a high technical threshold and focuses on increasing exercise enjoyment and compensatory psychological training. |
| Web-based Platform | A comprehensive health management portal accessed through a computer web browser. | Online course learning, health record access, and sophisticated risk assessment systems. | Suitable for static and in-depth information display. Interaction is primarily based on text input and clicks; while mobility is limited, it offers advantages in processing complex medical logic and large datasets. |
| Cardiac rehabilitation | Guideline-recommended comprehensive rehabilitation, typically conducted under the supervision of a medical institution. | Supervised exercise, face-to-face health education, and professional nutritional and psychological counseling. | In-person physical contact. Includes physical contact with professional staff and immediate safety supervision. |
| Usual care | Standard care and routine follow-up reminders received by patients upon hospital discharge. | Brief discharge guidance, printed educational materials, and irregular outpatient follow-up visits. | Typically one-way passive notification. Lacks continuous post-discharge supervision and dynamic intervention. |

Appendix 3: Risk of bias of randomized clinical trials

**Figure S3:** Overall risk of bias presented as percentage of each risk of bias item across all included studies. Green = Low risk, Red = High risk, Yellow = Some concerns.

**Table S3:** Study level risk of bias assessment using Cochrane risk of bias tool 2.0 for assessing risk of bias of randomized clinical trials.

| **Unique ID** | **Randomization process** | **Deviations from intended interventions** | **Mising outcome data** | **Measurement of the outcome** | **Selection of the reported result** | **Over all** |
| --- | --- | --- | --- | --- | --- | --- |
| An, 2015 | Low | Some concerns | Low | Some concerns | Some concerns | Some concerns |
| Avila, 2018 | Low | Low | Low | Some concerns | Low | Some concerns |
| Bai,2016 | Low | Some concerns | Low | Some concerns | Some concerns | Some concerns |
| Batalik, 2021 | Low | Some concerns | Some concerns | High | Low | High |
| Bernal-Jiménez, 2024 | Low | Some concerns | Low | Low | Low | Low |
| Boszko, 2023 | Low | Some concerns | Some concerns | Low | Low | Some concerns |
| Bretschneider, 2024 | Low | Some concerns | Some concerns | High | Low | High |
| Chen,2017 | Some concerns | Some concerns | Low | Some concerns | Some concerns | Some concerns |
| Cheung, 2023 | Low | Low | Low | Some concerns | Low | Some concerns |
| Chow 2022 | Low | Low | Low | Some concerns | Low | Some concerns |
| Dalli Peydró, 2022 | Low | Low | Low | Low | Low | Low |
| Dorje, 2019 | Low | Low | Low | Low | Low | Low |
| Ellis, 2025 | Low | Some concerns | Some concerns | Low | Low | Some concerns |
| Fu, 2025 | Some concerns | Some concerns | Low | Some concerns | Some concerns | Some concerns |
| Gu & Zhu 2023 | Low | Low | Some concerns | Some concerns | Some concerns | Some concerns |
| Hisam, 2022 | High | Some concerns | High | Low | Low | High |
| Hong, 2021 | Low | Low | Low | Some concerns | Low | Some concerns |
| Hua, 2024 | Some concerns | Some concerns | Low | Some concerns | Some concerns | Some concerns |
| Jóźwik,2021 | Low | High | High | Low | Low | Some concerns |
| Kraal, 2017 | Low | Low | Low | Some concerns | Low | Some concerns |
| Kumar, 2024 | Low | Low | Low | Low | Low | Low |
| Li 2024 | Low | Some concerns | Some concerns | Some concerns | Low | Some concerns |
| Liang,2023 | Some concerns | Some concerns | Low | Some concerns | Some concerns | Some concerns |
| Liu, 2026 | Low | Low | Low | Low | Low | Low |
| Ma 2021 | Low | Low | Some concerns | High | Some concerns | High |
| Maddison, 2019 | Low | Low | Low | Low | Low | Low |
| Pfaeffli Dale 2015 | Low | Low | Low | Some concerns | Low | Some concerns |
| Pitta, 2022 | Low | Some concerns | Low | Some concerns | Low | Some concerns |
| Reid, 2011 | Low | Low | Low | Low | Low | Low |
| Shariful Islam, 2019 | Low | Some concerns | Low | Some concerns | Low | Some concerns |
| Snoek 2019 | Low | Low | Low | High | Low | High |
| Su, 2021 | Low | Low | Some concerns | Low | Low | Some concerns |
| Vieira 2017 | Low | High | High | Some concerns | Low | High |
| Wei,2022 | Some concerns | Some concerns | Low | Some concerns | Some concerns | Some concerns |
| Zhang, 2025 | Low | Some concerns | Low | Some concerns | Low | Some concerns |
| Zhang,2020 | Some concerns | Some concerns | Low | Some concerns | Some concerns | Some concerns |
| Zhang,2024 | Some concerns | Some concerns | Low | Some concerns | Low | Some concerns |
| Zou, 2024 | Low | Low | Low | Low | Low | Low |
| Li 2025 | Low | Low | Low | Low | Low | Low |
| Gong Xue 2025 | Low | Low | Low | Some concerns | Some concerns | Some concerns |
| Zheng 2024 | Some concerns | High | Low | Some concerns | Some concerns | High |
| Fang 2018 | High | High | Some concerns | Some concerns | Some concerns | High |
| García-Bravo 2020 | Some concerns | Some concerns | Some concerns | Low | Low | Some concerns |
| Chong, 2025 | Low | Low | Some concerns | Low | Low | Low |

Appendix 4: Evaluation of inconsistency and heterogeneity

**Table S4.1:** Global consistency

| **Clinical outcome** | **Chi square** | **P value** | **Tau²** |
| --- | --- | --- | --- |
| Quality of life | 5.18 | 0.2694 | 0.45033225 |
| Anxiety | 2.86 | 0.2387 | 0.06685391 |
| Depression | 6.61 | 0.0667 | 0.11978521 |
| Adverse event | 1.29 | 0.9364 | 0.03247578 |

**Table S4.2:** Side-splitting of Quality of life.

| Comparison | | Direct | | Indirect | | Difference | | | tau |
| --- | --- | --- | --- | --- | --- | --- | --- | --- | --- |
|  |  | Coef. | Std.Err. | Coef. | Std.Err. | Coef. | Std.Err. | P>\|z\| |  |
| Mobile SMS | Usual care | 0.0106013 | 0.7447712 | -0.8247527 | 17.40897 | 0.835354 | 17.4249 | 0.962 | 1.05038 |
| Telephone Follow-up | Usual care | -2.97595 | 0.6241867 | 2.995098 | 115.4927 | -5.971048 | 115.4946 | 0.959 | 1.050339 |
| Mobile App | Usual care | -0.5517736 | 0.3788418 | 0.5704253 | 70.71934 | -1.122199 | 70.72044 | 0.987 | 1.050345 |
| Social Media | Cardiac rehabilitation | -3.405371 | 0.979802 | -0.5732834 | 0.6338385 | -2.832088 | 1.166946 | 0.153 | 0.9511596 |
| Social Media | Usual care | -0.8660671 | 0.3478825 | -3.696714 | 1.113726 | 2.830647 | 1.166793 | 0.157 | 0.9511536 |
| Wearables/Monitoring | Cardiac rehabilitation | -0.1208399 | 0.467025 | -1.881929 | 1.008461 | 1.761089 | 1.111418 | 0.113 | 1.01415 |
| Wearables/Monitoring | Usual care | -0.4451741 | 0.4671451 | 1.268214 | 1.01225 | -1.713389 | 1.114759 | 0.124 | 1.017175 |
| VR | Cardiac rehabilitation | -3.03E-07 | 1.157356 | -0.621277 | 1.277424 | 0.6212767 | 1.723745 | 0.719 | 1.067461 |
| VR | Usual care | -0.2713616 | 1.150401 | 0.349896 | 1.283651 | -0.6212577 | 1.723716 | 0.719 | 1.06746 |
| Web-based Platform | Usual care | -1.199356 | 0.7658755 | 1.217779 | 141.4614 | -2.417135 | 141.4637 | 0.986 | 1.050338 |
| Cardiac rehabilitation | Usual care | -0.1544965 | 1.100148 | 0.4155302 | 0.5982433 | -0.5700268 | 1.252288 | 0.649 | 1.066952 |

**Table S4.3:** Side-splitting of Anxiety.

| Comparison | | Direct | | Indirect | | Difference | | | tau |
| --- | --- | --- | --- | --- | --- | --- | --- | --- | --- |
|  |  | Coef. | Std.Err. | Coef. | Std.Err. | Coef. | Std.Err. | P>\|z\| |  |
| Mobile SMS | Cardiac rehabilitation | -0.134175 | 0.3338516 | -0.4289825 | 0.4138722 | 0.2948075 | 0.5317397 | 0.579 | 0.2772791 |
| Mobile SMS | Usual care | -0.0245351 | 0.2832172 | 0.2704807 | 0.4500035 | -0.2950158 | 0.5317097 | 0.579 | 0.2772792 |
| Telephone Follow-up | Usual care | 0.3221528 | 0.3226095 | -0.2114631 | 199.7916 | 0.5336159 | 199.792 | 0.998 | 0.2585616 |
| Mobile App | Usual care | 0.6080755 | 0.2017359 | -0.4968327 | 115.6129 | 1.104908 | 115.6132 | 0.992 | 0.2585615 |
| Social Media | Usual care | 0.3156747 | 0.1350395 | -0.204994 | 89.4404 | 0.5206686 | 89.44056 | 0.995 | 0.2585619 |
| Wearables/Monitoring | Cardiac rehabilitation | 0.237136 | 0.1966615 | -0.2086436 | 0.4502892 | 0.4457796 | 0.4902263 | 0.363 | 0.2650133 |
| Wearables/Monitoring | Usual care | 0.2784858 | 0.3232225 | 0.7242445 | 0.368576 | -0.4457587 | 0.4902256 | 0.363 | 0.2650134 |
| VR | Cardiac rehabilitation | 0.1768977 | 0.3224876 | 1.053225 | 0.3668274 | -0.8763278 | 0.4884268 | 0.093 | 0.2183101 |
| VR | Usual care | 1.120807 | 0.2707341 | 0.2444512 | 0.4062967 | 0.8763556 | 0.4884257 | 0.173 | 0.2183096 |
| Web-based Platform | Usual care | 1.564425 | 0.3945471 | -1.4538 | 200.0333 | 3.018226 | 200.0341 | 0.988 | 0.2585613 |

**Table S4.4:** Side-splitting of Depression.

| Comparison | | Direct | | Indirect | | Difference | | | tau |
| --- | --- | --- | --- | --- | --- | --- | --- | --- | --- |
|  |  | Coef. | Std.Err. | Coef. | Std.Err. | Coef. | Std.Err. | P>\|z\| |  |
| Mobile SMS | Cardiac rehabilitation | -0.4876235 | 0.3803386 | 0.1752131 | 0.3464184 | -0.6628367 | 0.5144542 | 0.198 | 0.3303109 |
| Mobile SMS | Usual care | 0.2541063 | 0.1959412 | -0.4083863 | 0.4756313 | 0.6624926 | 0.5143904 | 0.198 | 0.3303105 |
| Telephone Follow-up | Usual care | 0.3380475 | 0.3963729 | -0.0245006 | 200.0281 | 0.3625481 | 200.0287 | 0.999 | 0.3461768 |
| Mobile App | Usual care | 0.4735509 | 0.2404187 | -0.1599988 | 115.4451 | 0.6335497 | 115.4455 | 0.996 | 0.346177 |
| Social Media | Usual care | 0.5068823 | 0.170271 | -0.1933321 | 89.37983 | 0.7002144 | 89.38005 | 0.994 | 0.3461771 |
| Wearables/Monitoring | Cardiac rehabilitation | 0.2105483 | 0.2030971 | -0.9182194 | 0.3765985 | 1.128768 | 0.4275223 | 0.182 | 0.2800018 |
| Wearables/Monitoring | Usual care | -0.1522713 | 0.2492544 | 0.9764805 | 0.3473411 | -1.128752 | 0.4275197 | 0.086 | 0.2800017 |
| VR | Cardiac rehabilitation | 0.1063077 | 0.3390621 | 0.823044 | 0.435885 | -0.7167363 | 0.5517819 | 0.194 | 0.3425462 |
| VR | Usual care | 0.9175928 | 0.3319648 | 0.2008331 | 0.4422429 | 0.7167597 | 0.5517796 | 0.194 | 0.3425463 |
| Web-based Platform | Usual care | 1.071567 | 0.4437295 | -0.7581893 | 199.9924 | 1.829756 | 199.9933 | 0.993 | 0.3461771 |

**Table S4.5:** Side-splitting of Adverse event.

| Comparison | | Direct | | Indirect | | Difference | | | tau |
| --- | --- | --- | --- | --- | --- | --- | --- | --- | --- |
|  |  | Coef. | Std.Err. | Coef. | Std.Err. | Coef. | Std.Err. | P>\|z\| |  |
| Mobile SMS | Cardiac rehabilitation | -0.542763 | 0.6011753 | -0.4521231 | 0.5423232 | -0.0906399 | 0.8096457 | 0.911 | 1.39E-07 |
| Mobile SMS | Usual care | -0.0395956 | 0.1239224 | -0.1283635 | 0.7994175 | 0.0887679 | 0.8089655 | 0.913 | 4.71E-10 |
| Telephone Follow-up | Usual care | 1.177314 | 0.5458086 | -1.260692 | 115.4712 | 2.438006 | 115.4751 | 0.983 | 2.57E-08 |
| Mobile App | Usual care | 1.101635 | 0.3131438 | -1.185013 | 81.61389 | 2.286648 | 81.61569 | 0.978 | 5.20E-10 |
| Social Media | Cardiac rehabilitation | 0.7043646 | 1.233913 | 0.4093517 | 0.4860993 | 0.2950129 | 1.326207 | 0.824 | 1.84E-10 |
| Social Media | Usual care | 0.8903892 | 0.2408379 | 1.184532 | 1.303855 | -0.2941425 | 1.325926 | 0.824 | 9.00E-09 |
| Wearables/Monitoring | Cardiac rehabilitation | 0.1388487 | 0.4941002 | -0.186138 | 0.6745063 | 0.3249867 | 0.8361152 | 0.698 | 1.10E-09 |
| Wearables/Monitoring | Usual care | 0.4352939 | 0.3985112 | 0.6177806 | 0.7387044 | -0.1824866 | 0.8396695 | 0.828 | 1.13E-07 |
| VR | Cardiac rehabilitation | -0.0001406 | 2.046958 | 0.3807045 | 0.886324 | -0.3808451 | 2.230629 | 0.864 | 1.82E-10 |
| VR | Usual care | 0.8192175 | 0.7873339 | 0.4379308 | 2.086476 | 0.3812867 | 2.230145 | 0.864 | 1.45E-10 |
| Web-based Platform | Usual care | 0.567818 | 0.4400626 | -0.651045 | 141.4164 | 1.218863 | 141.4185 | 0.993 | 9.39E-07 |
| Cardiac rehabilitation | Usual care | 1.856986 | 1.495396 | 0.3460915 | 0.4144929 | 1.510894 | 1.548461 | 0.329 | 1.55E-09 |

Appendix 5: Network maps and forest plots of all outcomes

**Figure S5.1：**Network map of the effect on Quality of Life (QoL). The size of the nodes is proportional to the total number of participants randomized to each digital health intervention or control group, and the thickness of the lines connecting the nodes relates to the number of randomized controlled trials (RCTs) directly comparing those two specific interventions.

**Figure S5.2：**Network map of the effect on Anxiety scores. The size of the nodes is proportional to the total number of participants randomized to each digital health intervention or control group, and the thickness of the lines connecting the nodes relates to the number of randomized controlled trials (RCTs) directly comparing those specific interventions.

**Figure S5.3：**Network map of the effect on Depression scores. The size of the nodes is proportional to the total number of participants randomized to each digital health intervention or control group, and the thickness of the lines connecting the nodes relates to the number of randomized controlled trials (RCTs) directly comparing those specific interventions.

**Figure S5.4：**Network map of the effect on Adverse Events. The size of the nodes is proportional to the total number of participants randomized to each digital health intervention or control group, and the thickness of the lines connecting the nodes relates to the number of randomized controlled trials (RCTs) directly comparing those specific interventions.

Appendix 6: SUCRA and cumulative probability plots

**Figure S6.1:** Quality of Life (QoL)

**Table S6.1:** Quality of Life (QoL)

| **Treatment** | **SUCRA** | **PrBest** | **MeanRank** |
| --- | --- | --- | --- |
| **Mobile SMS** | 32.1 | 0 | 6.4 |
| **Telephone Follow-up** | 99.5 | 95.9 | 1 |
| **Mobile App** | 55.6 | 0 | 4.6 |
| **Social Media** | 76.3 | 0.4 | 2.9 |
| **Wearables/Monitoring** | 37.5 | 0 | 6 |
| **VR** | 32.2 | 0.1 | 6.4 |
| **Web-based Platform** | 72.2 | 3.5 | 3.2 |
| **Cardiac rehabilitation** | 17 | 0 | 7.6 |
| **Usual care** | 27.6 | 0 | 6.8 |

**Figure S6.2:**Anxiety scores

**Table S6.2:** Anxiety scores

| **Treatment** | **SUCRA** | **PrBest** | **MeanRank** |
| --- | --- | --- | --- |
| **Mobile SMS** | 15.8 | 0 | 7.7 |
| **Telephone Follow-up** | 41.5 | 0.6 | 5.7 |
| **Mobile App** | 67.1 | 0.6 | 3.6 |
| **Social Media** | 40.7 | 0 | 5.7 |
| **Wearables/Monitoring** | 56.6 | 0.3 | 4.5 |
| **VR** | 82.7 | 6.1 | 2.4 |
| **Web-based Platform** | 98.6 | 92.4 | 1.1 |
| **Cardiac rehabilitation** | 38.3 | 0 | 5.9 |
| **Usual care** | 8.6 | 0 | 8.3 |

**Figure S6.3:** Depression scores

**Table S6.3:** Depression scores

| **Treatment** | **SUCRA** | **PrBest** | **MeanRank** |
| --- | --- | --- | --- |
| **Mobile SMS** | 26.9 | 0.1 | 6.8 |
| **Telephone Follow-up** | 46.2 | 5.6 | 5.3 |
| **Mobile App** | 59.5 | 4.3 | 4.2 |
| **Social Media** | 64.7 | 3.4 | 3.8 |
| **Wearables/Monitoring** | 34.9 | 0.3 | 6.2 |
| **VR** | 76.5 | 14.5 | 2.9 |
| **Web-based Platform** | 91.4 | 71.5 | 1.7 |
| **Cardiac rehabilitation** | 40.8 | 0.3 | 5.7 |
| **Usual care** | 9 | 0 | 8.3 |

**Figure S6.3:** Adverse Events

**Table S6.4:** Adverse Events

| **Treatment** | **SUCRA** | **PrBest** | **MeanRank** |
| --- | --- | --- | --- |
| **Mobile SMS** | 10.2 | 0 | 8.2 |
| **Telephone Follow-up** | 81.1 | 39.3 | 2.6 |
| **Mobile App** | 79.6 | 26.2 | 2.5 |
| **Social Media** | 70.7 | 7.7 | 3.3 |
| **Wearables/Monitoring** | 43.9 | 0.7 | 5.5 |
| **VR** | 58.5 | 20.4 | 4.3 |
| **Web-based Platform** | 49.8 | 4.1 | 5 |
| **Cardiac rehabilitation** | 14.3 | 0 | 7.9 |
| **Usual care** | 41.9 | 1.6 | 5.6 |

Appendix 7:Summary Ranking Table of the Effects of Digital Health Interventions.

**Table S7.1:** Quality of Life (QoL)

**Table S7.2:**Anxiety scores

**Table S7.3:**Depression scores

**Table S7.4:** Adverse Events

† Note: The TEXTMEDS trial (Chow 2022), while primarily classified as an SMS-based intervention, incorporated a 2-way communication option and a dedicated health counselor who monitored patient replies. This heterogeneity is acknowledged and addressed in the mechanistic interpretation of the safety findings (see Results section ‘Incidence of Adverse Events’), where we note that the inclusion of this partially interactive SMS trial would likely dilute—rather than exaggerate—the observed safety risk estimate for the SMS node.

Appendix 8: CINeMA Assessment

We use the CINeMA framework to evidence certainty, assessing it for each network estimate based on the following criteria:

**Figure S8.1:** Risk of bias contribution by intervention group in Quality of life.

**Figure S8.2:** Overall risk of bias by treatment comparison in Quality of life.

**Figure S8.3:** Risk of bias contribution by intervention group in Anxiety Scores.

**Figure S8.4:** Overall risk of bias by treatment comparison in Anxiety Scores.

**Figure S8.5:** Risk of bias contribution by intervention group in Depression Scores.

**Figure S8.6:** Overall risk of bias by treatment comparison in Depression Scores.

**Figure S8.7:** Risk of bias contribution by intervention group in Adverse Events.

**Figure S8.8:** Overall risk of bias by treatment comparison in Adverse Events.

**Table S8.1:** Transitivity (Indirectness) Assessment

|  | Baseline variable (Mean ± SD) | |
| --- | --- | --- |
| Intervention | Age(year) | Male % |
| Mobile SMS | 58.57 ± 10.26 | 78.6% |
| Telephone Follow-up | 59.31 ± 11.80 | 59.8% |
| Mobile App | 60.42 ± 9.03 | 74.7% |
| Social Media | 60.73 ± 8.76 | 73.3% |
| Wearables/Monitoring | 60.19 ± 10.38 | 80.9% |
| VR | 57.60 ± 11.17 | 52.8% |
| Web-based Platform | 60.14 ± 10.79 | 77.2% |
| Cardiac rehabilitation | 60.26 ± 10.26 | 81.9% |
| Usual care | 59.77 ± 10.32 | 78.5% |

**Imprecision:** We use the CINeMA website to grade the accuracy of each comparison.

**Heterogeneity:** We assessed the degree of worry by comparing clinical reasoning based on 95% confidence intervals (CIs) while applying the same clinical reasoning framework as for inaccuracy. In particular, we judged the consistency of our findings based on the confidence and prediction intervals associated with clinically important effect sizes. And we used the same thresholds of clinical significance as described above and followed the recommendations automatically provided by CINeMA (<https://cinema.ispm.unibe.ch/>).

**Inconsistency:** For inconsistency, we looked at the results for node splitting and we saw major problems when p<0.10, but otherwise no problems.

**Table S8.2:**CINeMA Results of Quality of life

| Comparison | Within-study bias | Reporting bias | Indirectness | Imprecision | Heterogeneity | Incoherence | Confidence rating |
| --- | --- | --- | --- | --- | --- | --- | --- |
| Cardiac rehabilitation:Social Media | No concerns | Low risk | No concerns | No concerns | Major concerns | Major concerns | Very low |
| Cardiac rehabilitation:Usual care | Some concerns | Low risk | No concerns | Major concerns | No concerns | No concerns | Low |
| Cardiac rehabilitation:VR | Some concerns | Low risk | No concerns | Major concerns | No concerns | No concerns | Low |
| Cardiac rehabilitation:Wearables/Monitoring | Some concerns | Low risk | No concerns | Major concerns | No concerns | Major concerns | Very low |
| Mobile App:Usual care | Some concerns | Low risk | No concerns | Major concerns | No concerns | Major concerns | Very low |
| Mobile SMS:Usual care | Some concerns | Low risk | No concerns | Major concerns | No concerns | Major concerns | Very low |
| Social Media:Usual care | Some concerns | Low risk | No concerns | No concerns | Major concerns | No concerns | Moderate |
| Telephone Follow-up:Usual care | Some concerns | Low risk | No concerns | No concerns | No concerns | Major concerns | Moderate |
| Usual care:VR | Some concerns | Low risk | No concerns | Major concerns | No concerns | No concerns | Low |
| Usual care:Wearables/Monitoring | Major concerns | Low risk | No concerns | Major concerns | No concerns | No concerns | Very low |
| Usual care:Web-based Platform | No concerns | Low risk | No concerns | No concerns | Major concerns | Major concerns | Low |
| Cardiac rehabilitation:Mobile App | Some concerns | Low risk | No concerns | Major concerns | No concerns | Major concerns | Very low |
| Cardiac rehabilitation:Mobile SMS | Some concerns | Low risk | No concerns | Major concerns | No concerns | Major concerns | Very low |
| Cardiac rehabilitation:Telephone Follow-up | Some concerns | Low risk | No concerns | No concerns | No concerns | Major concerns | Moderate |
| Cardiac rehabilitation:Web-based Platform | Some concerns | Low risk | No concerns | No concerns | Major concerns | Major concerns | Low |
| Mobile App:Mobile SMS | Some concerns | Low risk | No concerns | Major concerns | No concerns | Major concerns | Very low |
| Mobile App:Social Media | Some concerns | Low risk | No concerns | Major concerns | No concerns | Major concerns | Very low |
| Mobile App:Telephone Follow-up | Some concerns | Low risk | No concerns | No concerns | No concerns | Major concerns | Moderate |
| Mobile App:VR | Some concerns | Low risk | No concerns | Major concerns | No concerns | Major concerns | Very low |
| Mobile App:Wearables/Monitoring | Some concerns | Low risk | No concerns | Major concerns | No concerns | Major concerns | Very low |
| Mobile App:Web-based Platform | Some concerns | Low risk | No concerns | Major concerns | No concerns | Major concerns | Very low |
| Mobile SMS:Social Media | Some concerns | Low risk | No concerns | Major concerns | No concerns | Major concerns | Very low |
| Mobile SMS:Telephone Follow-up | Some concerns | Low risk | No concerns | No concerns | No concerns | Major concerns | Moderate |
| Mobile SMS:VR | Some concerns | Low risk | No concerns | Major concerns | No concerns | Major concerns | Very low |
| Mobile SMS:Wearables/Monitoring | Some concerns | Low risk | No concerns | Major concerns | No concerns | Major concerns | Very low |
| Mobile SMS:Web-based Platform | Some concerns | Low risk | No concerns | Major concerns | No concerns | Major concerns | Very low |
| Social Media:Telephone Follow-up | Some concerns | Low risk | No concerns | No concerns | Major concerns | Major concerns | Low |
| Social Media:VR | Some concerns | Low risk | No concerns | Major concerns | No concerns | Major concerns | Very low |
| Social Media:Wearables/Monitoring | Some concerns | Low risk | No concerns | No concerns | Major concerns | Major concerns | Low |
| Social Media:Web-based Platform | Some concerns | Low risk | No concerns | Major concerns | No concerns | Major concerns | Very low |
| Telephone Follow-up:VR | Some concerns | Low risk | No concerns | No concerns | No concerns | Major concerns | Moderate |
| Telephone Follow-up:Wearables/Monitoring | Some concerns | Low risk | No concerns | No concerns | No concerns | Major concerns | Moderate |
| Telephone Follow-up:Web-based Platform | Some concerns | Low risk | No concerns | No concerns | Major concerns | Major concerns | Low |
| VR:Wearables/Monitoring | Some concerns | Low risk | No concerns | Major concerns | No concerns | Major concerns | Very low |
| VR:Web-based Platform | Some concerns | Low risk | No concerns | Major concerns | No concerns | Major concerns | Very low |
| Wearables/Monitoring:Web-based Platform | Some concerns | Low risk | No concerns | Major concerns | No concerns | Major concerns | Very low |

**Table S8.3:**CINeMA Results of Anxiety Scores

| Comparison | Within-study bias | Reporting bias | Indirectness | Imprecision | Heterogeneity | Incoherence | Confidence rating |
| --- | --- | --- | --- | --- | --- | --- | --- |
| Cardiac rehabilitation:Mobile SMS | Some concerns | Low risk | No concerns | Major concerns | No concerns | No concerns | Low |
| Cardiac rehabilitation:VR | Some concerns | Low risk | No concerns | No concerns | Major concerns | Major concerns | Very low |
| Cardiac rehabilitation:Wearables/Monitoring | No concerns | Low risk | No concerns | Major concerns | No concerns | No concerns | Low |
| Mobile App:Usual care | Some concerns | Low risk | No concerns | No concerns | No concerns | Major concerns | Moderate |
| Mobile SMS:Usual care | Some concerns | Low risk | No concerns | Major concerns | No concerns | No concerns | Low |
| Social Media:Usual care | No concerns | Low risk | No concerns | No concerns | Major concerns | Major concerns | Low |
| Telephone Follow-up:Usual care | Some concerns | Low risk | No concerns | Major concerns | No concerns | Major concerns | Very low |
| Usual care:VR | Some concerns | Low risk | No concerns | No concerns | No concerns | Major concerns | Moderate |
| Usual care:Wearables/Monitoring | Some concerns | Low risk | No concerns | No concerns | Major concerns | No concerns | Moderate |
| Usual care:Web-based Platform | No concerns | Low risk | No concerns | No concerns | No concerns | Major concerns | Moderate |
| Cardiac rehabilitation:Mobile App | Some concerns | Low risk | No concerns | Major concerns | No concerns | Major concerns | Very low |
| Cardiac rehabilitation:Social Media | Some concerns | Low risk | No concerns | Major concerns | No concerns | Major concerns | Very low |
| Cardiac rehabilitation:Telephone Follow-up | Some concerns | Low risk | No concerns | Major concerns | No concerns | Major concerns | Very low |
| Cardiac rehabilitation:Usual care | Some concerns | Low risk | No concerns | Major concerns | No concerns | Major concerns | Very low |
| Cardiac rehabilitation:Web-based Platform | Some concerns | Low risk | No concerns | No concerns | No concerns | Major concerns | Moderate |
| Mobile App:Mobile SMS | Some concerns | Low risk | No concerns | No concerns | Major concerns | Major concerns | Low |
| Mobile App:Social Media | Some concerns | Low risk | No concerns | Major concerns | No concerns | Major concerns | Very low |
| Mobile App:Telephone Follow-up | Some concerns | Low risk | No concerns | Major concerns | No concerns | Major concerns | Very low |
| Mobile App:VR | Some concerns | Low risk | No concerns | Major concerns | No concerns | Major concerns | Very low |
| Mobile App:Wearables/Monitoring | Some concerns | Low risk | No concerns | Major concerns | No concerns | Major concerns | Very low |
| Mobile App:Web-based Platform | Some concerns | Low risk | No concerns | No concerns | No concerns | Major concerns | Moderate |
| Mobile SMS:Social Media | Some concerns | Low risk | No concerns | Major concerns | No concerns | Major concerns | Very low |
| Mobile SMS:Telephone Follow-up | Some concerns | Low risk | No concerns | Major concerns | No concerns | Major concerns | Very low |
| Mobile SMS:VR | Some concerns | Low risk | No concerns | No concerns | No concerns | Major concerns | Moderate |
| Mobile SMS:Wearables/Monitoring | Some concerns | Low risk | No concerns | Major concerns | No concerns | Major concerns | Very low |
| Mobile SMS:Web-based Platform | Some concerns | Low risk | No concerns | No concerns | No concerns | Major concerns | Moderate |
| Social Media:Telephone Follow-up | Some concerns | Low risk | No concerns | Major concerns | No concerns | Major concerns | Very low |
| Social Media:VR | Some concerns | Low risk | No concerns | No concerns | Major concerns | Major concerns | Low |
| Social Media:Wearables/Monitoring | Some concerns | Low risk | No concerns | Major concerns | No concerns | Major concerns | Very low |
| Social Media:Web-based Platform | No concerns | Low risk | No concerns | No concerns | No concerns | Major concerns | Moderate |
| Telephone Follow-up:VR | Some concerns | Low risk | No concerns | Major concerns | No concerns | Major concerns | Very low |
| Telephone Follow-up:Wearables/Monitoring | Some concerns | Low risk | No concerns | Major concerns | No concerns | Major concerns | Very low |
| Telephone Follow-up:Web-based Platform | Some concerns | Low risk | No concerns | No concerns | No concerns | Major concerns | Moderate |
| VR:Wearables/Monitoring | Some concerns | Low risk | No concerns | Major concerns | No concerns | Major concerns | Very low |
| VR:Web-based Platform | Some concerns | Low risk | No concerns | No concerns | Major concerns | Major concerns | Low |
| Wearables/Monitoring:Web-based Platform | Some concerns | Low risk | No concerns | No concerns | No concerns | Major concerns | Moderate |

**Table S8.4:**CINeMA Results of Depression Scores

| Comparison | Within-study bias | Reporting bias | Indirectness | Imprecision | Heterogeneity | Incoherence | Confidence rating |
| --- | --- | --- | --- | --- | --- | --- | --- |
| Cardiac rehabilitation:Mobile SMS | Some concerns | Low risk | No concerns | Major concerns | No concerns | No concerns | Low |
| Cardiac rehabilitation:VR | Some concerns | Low risk | No concerns | Major concerns | No concerns | No concerns | Low |
| Cardiac rehabilitation:Wearables/Monitoring | No concerns | Low risk | No concerns | Major concerns | No concerns | Major concerns | Very low |
| Mobile App:Usual care | Some concerns | Low risk | No concerns | No concerns | Major concerns | Major concerns | Low |
| Mobile SMS:Usual care | Some concerns | Low risk | No concerns | Major concerns | No concerns | No concerns | Low |
| Social Media:Usual care | No concerns | Low risk | No concerns | No concerns | Major concerns | Major concerns | Low |
| Telephone Follow-up:Usual care | Some concerns | Low risk | No concerns | Major concerns | No concerns | Major concerns | Very low |
| Usual care:VR | Some concerns | Low risk | No concerns | No concerns | Major concerns | No concerns | Moderate |
| Usual care:Wearables/Monitoring | Major concerns | Low risk | No concerns | Major concerns | No concerns | Major concerns | Very low |
| Usual care:Web-based Platform | Some concerns | Low risk | No concerns | No concerns | Major concerns | Major concerns | Low |
| Cardiac rehabilitation:Mobile App | Some concerns | Low risk | No concerns | Major concerns | No concerns | Major concerns | Very low |
| Cardiac rehabilitation:Social Media | Some concerns | Low risk | No concerns | Major concerns | No concerns | Major concerns | Very low |
| Cardiac rehabilitation:Telephone Follow-up | Some concerns | Low risk | No concerns | Major concerns | No concerns | Major concerns | Very low |
| Cardiac rehabilitation:Usual care | Some concerns | Low risk | No concerns | Major concerns | No concerns | Major concerns | Very low |
| Cardiac rehabilitation:Web-based Platform | Some concerns | Low risk | No concerns | Major concerns | No concerns | Major concerns | Very low |
| Mobile App:Mobile SMS | Some concerns | Low risk | No concerns | Major concerns | No concerns | Major concerns | Very low |
| Mobile App:Social Media | Some concerns | Low risk | No concerns | Major concerns | No concerns | Major concerns | Very low |
| Mobile App:Telephone Follow-up | Some concerns | Low risk | No concerns | Major concerns | No concerns | Major concerns | Very low |
| Mobile App:VR | Some concerns | Low risk | No concerns | Major concerns | No concerns | Major concerns | Very low |
| Mobile App:Wearables/Monitoring | Some concerns | Low risk | No concerns | Major concerns | No concerns | Major concerns | Very low |
| Mobile App:Web-based Platform | Some concerns | Low risk | No concerns | Major concerns | No concerns | Major concerns | Very low |
| Mobile SMS:Social Media | Some concerns | Low risk | No concerns | Major concerns | No concerns | Major concerns | Very low |
| Mobile SMS:Telephone Follow-up | Some concerns | Low risk | No concerns | Major concerns | No concerns | Major concerns | Very low |
| Mobile SMS:VR | Some concerns | Low risk | No concerns | Major concerns | No concerns | Major concerns | Very low |
| Mobile SMS:Wearables/Monitoring | Some concerns | Low risk | No concerns | Major concerns | No concerns | Major concerns | Very low |
| Mobile SMS:Web-based Platform | Some concerns | Low risk | No concerns | Major concerns | No concerns | Major concerns | Very low |
| Social Media:Telephone Follow-up | Some concerns | Low risk | No concerns | Major concerns | No concerns | Major concerns | Very low |
| Social Media:VR | Some concerns | Low risk | No concerns | Major concerns | No concerns | Major concerns | Very low |
| Social Media:Wearables/Monitoring | Major concerns | Low risk | No concerns | Major concerns | No concerns | Major concerns | Very low |
| Social Media:Web-based Platform | Some concerns | Low risk | No concerns | Major concerns | No concerns | Major concerns | Very low |
| Telephone Follow-up:VR | Some concerns | Low risk | No concerns | Major concerns | No concerns | Major concerns | Very low |
| Telephone Follow-up:Wearables/Monitoring | Some concerns | Low risk | No concerns | Major concerns | No concerns | Major concerns | Very low |
| Telephone Follow-up:Web-based Platform | Some concerns | Low risk | No concerns | Major concerns | No concerns | Major concerns | Very low |
| VR:Wearables/Monitoring | Some concerns | Low risk | No concerns | Major concerns | No concerns | Major concerns | Very low |
| VR:Web-based Platform | Some concerns | Low risk | No concerns | Major concerns | No concerns | Major concerns | Very low |
| Wearables/Monitoring:Web-based Platform | Some concerns | Low risk | No concerns | Major concerns | No concerns | Major concerns | Very low |

**Table S8.5:**CINeMA Results of Adverse Events

| Comparison | Within-study bias | Reporting bias | Indirectness | Imprecision | Heterogeneity | Incoherence | Confidence rating |
| --- | --- | --- | --- | --- | --- | --- | --- |
| Cardiac rehabilitation:Mobile SMS | Some concerns | Low risk | No concerns | Major concerns | No concerns | No concerns | Low |
| Cardiac rehabilitation:Social Media | Some concerns | Low risk | No concerns | Major concerns | No concerns | No concerns | Low |
| Cardiac rehabilitation:Usual care | Some concerns | Low risk | No concerns | Major concerns | No concerns | No concerns | Low |
| Cardiac rehabilitation:VR | Some concerns | Low risk | No concerns | Major concerns | No concerns | No concerns | Low |
| Cardiac rehabilitation:Wearables/Monitoring | Some concerns | Low risk | No concerns | Major concerns | No concerns | No concerns | Low |
| Mobile App:Usual care | Major concerns | Low risk | No concerns | No concerns | No concerns | No concerns | Moderate |
| Mobile SMS:Usual care | Some concerns | Low risk | No concerns | Major concerns | No concerns | No concerns | Low |
| Social Media:Usual care | Some concerns | Low risk | No concerns | No concerns | No concerns | No concerns | High |
| Telephone Follow-up:Usual care | Some concerns | Low risk | No concerns | No concerns | No concerns | No concerns | High |
| Usual care:VR | Some concerns | Low risk | No concerns | Major concerns | No concerns | No concerns | Low |
| Usual care:Wearables/Monitoring | Major concerns | Low risk | No concerns | Major concerns | No concerns | No concerns | Very low |
| Usual care:Web-based Platform | No concerns | Low risk | No concerns | Major concerns | No concerns | No concerns | Low |
| Cardiac rehabilitation:Mobile App | Some concerns | Low risk | No concerns | Major concerns | No concerns | No concerns | Low |
| Cardiac rehabilitation:Telephone Follow-up | Some concerns | Low risk | No concerns | Major concerns | No concerns | No concerns | Low |
| Cardiac rehabilitation:Web-based Platform | Some concerns | Low risk | No concerns | Major concerns | No concerns | No concerns | Low |
| Mobile App:Mobile SMS | Some concerns | Low risk | No concerns | No concerns | No concerns | No concerns | High |
| Mobile App:Social Media | Major concerns | Low risk | No concerns | Major concerns | No concerns | No concerns | Very low |
| Mobile App:Telephone Follow-up | Some concerns | Low risk | No concerns | Major concerns | No concerns | No concerns | Low |
| Mobile App:VR | Some concerns | Low risk | No concerns | Major concerns | No concerns | No concerns | Low |
| Mobile App:Wearables/Monitoring | Major concerns | Low risk | No concerns | Major concerns | No concerns | No concerns | Very low |
| Mobile App:Web-based Platform | No concerns | Low risk | No concerns | Major concerns | No concerns | No concerns | Low |
| Mobile SMS:Social Media | Some concerns | Low risk | No concerns | No concerns | No concerns | No concerns | High |
| Mobile SMS:Telephone Follow-up | Some concerns | Low risk | No concerns | No concerns | No concerns | No concerns | High |
| Mobile SMS:VR | Some concerns | Low risk | No concerns | Major concerns | No concerns | No concerns | Low |
| Mobile SMS:Wearables/Monitoring | Some concerns | Low risk | No concerns | Major concerns | No concerns | No concerns | Low |
| Mobile SMS:Web-based Platform | Some concerns | Low risk | No concerns | Major concerns | No concerns | No concerns | Low |
| Social Media:Telephone Follow-up | Some concerns | Low risk | No concerns | Major concerns | No concerns | No concerns | Low |
| Social Media:VR | Some concerns | Low risk | No concerns | Major concerns | No concerns | No concerns | Low |
| Social Media:Wearables/Monitoring | Major concerns | Low risk | No concerns | Major concerns | No concerns | No concerns | Very low |
| Social Media:Web-based Platform | Some concerns | Low risk | No concerns | Major concerns | No concerns | No concerns | Low |
| Telephone Follow-up:VR | Some concerns | Low risk | No concerns | Major concerns | No concerns | No concerns | Low |
| Telephone Follow-up:Wearables/Monitoring | Some concerns | Low risk | No concerns | Major concerns | No concerns | No concerns | Low |
| Telephone Follow-up:Web-based Platform | Some concerns | Low risk | No concerns | Major concerns | No concerns | No concerns | Low |
| VR:Wearables/Monitoring | Some concerns | Low risk | No concerns | Major concerns | No concerns | No concerns | Low |
| VR:Web-based Platform | Some concerns | Low risk | No concerns | Major concerns | No concerns | No concerns | Low |
| Wearables/Monitoring:Web-based Platform | Major concerns | Low risk | No concerns | Major concerns | No concerns | No concerns | Very low |

Appendix 9: Funnel plots

The figures display the assessment of small-study effects and potential publication bias regarding the impact of various digital health interventions on clinical and psychological outcomes in patients with coronary heart disease. The funnel plots include all trials comparing at least one digital health intervention (e.g., Mobile App, VR, or Telephone Follow-up) against usual care, conventional cardiac rehabilitation (CR), or other active digital comparators. In the case of multi-arm trials—for instance, a study comparing Usual Care vs. Mobile App vs. Wearables—each pairwise comparison (e.g., Usual Care vs. Mobile App and Usual Care vs. Wearables) was plotted separately.

**Figure S9.1:** Funnel plot of Quality of Life (QoL)

**Figure S9.2:** Funnel plot of Anxiety scores

**Figure S9.3:** Funnel plot of Depression scores

**Figure S9.4:** Funnel plot of Adverse Events

Appendix 10: Sensitivity analyses

**Table S10.1:** Sensitivity analyses of primary outcomes

| Intervention Type | Quality of Life (QoL) | | Anxiety scores | | Depression scores | |
| --- | --- | --- | --- | --- | --- | --- |
|  | Main estimate | Sensitivity analyses | Main estimate | Sensitivity analyses | Main estimate | Sensitivity analyses |
| Mobile SMS | -0.00 (-1.45,1.44) | -0.00 (-1.62,1.61) | -0.06 (-0.50,0.39) | -0.14 (-0.69,0.41) | -0.16 (-0.53,0.21) | -0.27 (-0.61,0.07) |
| Telephone Follow-up | 2.98 (1.75,4.20) | 2.98(1.62,4.34) | -0.32 (-0.95,0.31) | -0.32 (-1.03,0.38) | -0.34 (-1.11,0.44) | -0.34 (-1.04,0.37) |
| Mobile App | 0.55 (-0.19,1.29) | 0.42 (-0.46,1.30) | -0.61 (-1.00,-0.21) | -0.61 (-1.05,-0.18) | -0.47 (-0.94,-0.00) | -0.47 (-0.90,-0.04) |
| Social Media | 1.13(0.42,1.84) | 1.25 (0.40,2.10) | -0.32 (-0.58,-0.05) | -0.30 (-0.64,-0.03) | -0.51 (-0.84,-0.17) | -0.55 (-0.89,-0.22) |
| Wearables/Monitoring | 0.15 (-0.71,1.00) | 0.14 (-1.25,1.28) | -0.47 (-0.94,-0.00) | -0.72 (-1.54,-0.09) | -0.23 (-0.69,0.23) | -1.03 (-1.77,-0.29) |
| VR | -0.01 (-1.66,1.65) | -0.52 (-3.36,2.33) | -0.86 (-1.34,-0.38) | -0.93 (-1.56,-0.30) | -0.66 (-1.18,-0.13) | -0.99 (-1.59,-0.40) |
| Web-based Platform | 1.20 (-0.30,2.70) | 1.21 (-0.46,2.87) | -1.56 (-2.34,-0.79) | -1.56 (-2.40,-0.73) | -1.07 (-1.94,-0.20) | -1.07 (-1.88,-0.26) |
| Cardiac rehabilitation | -0.29 (-1.30,0.73) | -0.52 (-1.95,0.91) | -0.30 (-0.77,0.16) | -0.49 (-1.19,0.20) | -0.28 (-0.76,0.20) | -0.82 (-1.43,-0.21) |

Appendix 11: Subgroup analyses based on different intervention contents

**Table S11.1：**Classification list of digital intervention contents

| **Interventions and Controls** | **Content definitions** | **Intervention mechanisms** |
| --- | --- | --- |
| Health Education | Based on the patient's specific health status, disease stage, or demographic characteristics, the system unidirectionally delivers evidence-based health information regarding disease management, treatment side effects, and daily care. | Fills the disease knowledge gap and enhances patients' cognition and self-efficacy regarding cardiovascular diseases, thereby alleviating the anxiety and uncertainty caused by unknown disease progression and prognosis. |
| Health Tracking | Empowers patients with the ability to self-record, monitor, and report their physiological data (e.g., heart rate, blood pressure) and subjective symptoms (e.g., pain, fatigue). | Achieves early identification of cardiovascular risks and provides timely clinical feedback through continuous data monitoring and dynamic early warnings, thereby enhancing patients' sense of disease control and security. |
| Psychological Intervention | Focuses on the remodeling of patients' cognition, emotion, and mental health. It utilizes digital platforms to provide structured psychological therapy or counseling, such as web-based cognitive behavioral therapy (CBT), mindfulness-based stress reduction, expert online psychological consultation, or peer support networks. | Directly targets cardiac anxiety and emotional disorders, and corrects patients' excessive attention to cardiac symptoms and catastrophic thinking through cognitive restructuring, effectively reducing depression and anxiety scores. |
| Exercise Intervention | Digital interventions focusing on improving patients' physical function, physical endurance, and specific organ rehabilitation. | Improves cardiopulmonary fitness and vascular endothelial function through exercise mediation, and indirectly alleviates coronary heart disease-related somatic fatigue and depressive symptoms through physiological mechanisms such as endorphin release. |
| Psychological & Exercise Combined | Interventions that simultaneously integrate the dual active components of "structured psychological support" and "physical exercise rehabilitation" within the same digital health tool. | Provides simultaneous treatment for both mental and physical "dual targets"; psychological interventions help overcome patients' fear of exercise-induced cardiac events, while exercise improves overall physical fitness, generating a synergistic effect in improving mood and quality of life (QoL). |
| Cardiac rehabilitation（CR） | A comprehensive program supervised face-to-face by a multidisciplinary team in a hospital or specialized rehabilitation center, encompassing exercise training, risk factor management, and psychological assessment. | Relies on the direct supervision and immediate interpersonal emotional support of medical professionals to systematically optimize cardiovascular risk factors and ensure the absolute safety of patients during exercise. |
| Usual care | Routine cardiology outpatient follow-ups, basic discharge medical advice, and standard secondary prevention medication for coronary heart disease. | Relies on patients' self-motivation and the basic pharmacological effects of medications, lacking continuous behavioral change triggering mechanisms and professional remote real-time feedback. |

**Figure S11.1：**Effects of different intervention contents

Network meta-analysis plots illustrating the efficacy of different intervention contents on various outcomes in patients with coronary heart disease: (A) Quality of life, (B) Anxiety Scores, and (C) Depression Scores. Node size reflects the number of study participants for each intervention type, and the thickness of the lines connecting interventions indicates the number of studies involved in that comparison.

(A1) Network map of Quality of life.

(A2) Forest plot of Quality of life.

(B1) Network map of Anxiety Scores.

(B2) Forest plot of Anxiety Scores.

(C1) Network map of Depression Scores.

(C2) Forest plot of Depression Scores.

Appendix 12:Impact of intervention duration on outcome measures

**Figure S12.1:**The effects of digital health interventions in short-term interventions (＜6 months)

(A1) Forest plot of Quality of life.

(B1) Forest plot of Anxiety Scores.

(C1) Forest plot of Depression Scores.

(D1)Forest plot of Retention rates.

**Figure S12.2:**The effects of digital health interventions in long-term interventions (≥6 months)

(A) Forest plot of Quality of life.

(B) Forest plot of Anxiety Scores.

(C) Forest plot of Depression Scores.

(D)Forest plot of Retention rates.

**Appendix13:Comparison of the effectiveness of intervention measures across countries with different income levels**

**TableS13:** Comparison of outcomes across different digital health interventions

| **Classification** | **Income Level** | **Quality of life** | **Anxiety** | **Depression** |
| --- | --- | --- | --- | --- |
| Mobile SMS | High-income | -0.01 (-0.41,0.39) | NA | NA |
|  | Middle-income | NA | NA | NA |
| Telephone Follow-up | High-income | NA | NA | NA |
|  | Middle-income | 2.98 (1.57,4.39) | -0.32 (-0.72,0.08) | -0.34 (-0.79,0.11) |
| Mobile App | High-income | 0.30 (-0.09,0.70) | NA | NA |
|  | Middle-income | 0.70 (-0.39,1.79) | -0.60 (-0.96,-0.24) | -0.47 (-0.86,-0.07) |
| Social Media | High-income | NA | NA | NA |
|  | Middle-income | 0.90 (0.04,1.77) | -0.31 (-0.55,-0.08) | -0.50 (-0.77,-0.24) |
| Wearables/ Monitoring | High-income | 0.21 (-0.24,0.67) | NA | NA |
|  | Middle-income | 0.61 (-0.80,2.02) | -0.28 (-0.84,0.28) | 0.32 (-0.39,1.02) |
| VR | High-income | 0.19 (-0.56,0.95) | NA | NA |
|  | Middle-income | 0.27 (-2.27,2.81) | -1.18 (-1.79,-0.56) | -1.06 (-1.74,-0.39) |
| Web-based Platform | High-income | 0.13 (-0.50,0.76) | NA | NA |
|  | Middle-income | 2.35 (-0.14,4.84) | -1.56 (-2.29,-0.84) | -1.07 (-1.82,-0.32) |
| Cardiac rehabilitation | High-income | 0.11 (-0.38,0.60) | NA | NA |
|  | Middle-income | -2.50 (-5.09,0.09) | 0.28 (-0.49,1.05) | 0.79 (-0.14,1.71) |

Note: Income levels are classified according to the World Bank lending groups (2024–2025).
